# Chemotherapy‐Activated GSK3β‐DNMT1 Signaling Upregulates CD47 to Evade Macrophage Phagocytosis and Drive Temozolomide Resistance in Glioblastoma

**DOI:** 10.1002/advs.77471

**Published:** 2026-08-29

**Authors:** Jie Li, Shuai Ying, Yi Zhang, Jingjing Wang, Quan Wan, Lingli Gong, Daxing Xu, Zhenkun Yang, Hongnian Lu, Ying Yin, Xiangming Fang, Zhening Pu

**Affiliations:** ^1^ Department of Laboratory Medicine The Affiliated Wuxi People's Hospital of Nanjing Medical University Wuxi People's Hospital Wuxi Medical Center of Nanjing Medical University Wuxi Jiangsu China; ^2^ Department of Radiology The Affiliated Wuxi People's Hospital of Nanjing Medical University Wuxi People's Hospital Wuxi Medical Center of Nanjing Medical University Wuxi Jiangsu China; ^3^ Neuroscience Center Wuxi School of Medicine Jiangnan University Wuxi Jiangsu China

**Keywords:** CD47, chemoresistance, DNA methylation, glioblastoma, tumor‐associated macrophages

## Abstract

Temozolomide (TMZ) remains the first‐line therapy for glioblastoma (GBM) patients. However, the mechanisms underlying the emergence of acquired TMZ resistance after treatment are unclear. Here, we reveal the critical role of macrophage phagocytosis in GBM recurrence and acquired TMZ resistance. Mechanistically, TMZ treatment sustained GSK3β activation and promoted its interaction with DNMT1. This led to the phosphorylation of DNMT1 at the previously unrecognized S977 site for its K981‐dependent ubiquitination and destabilization. The downregulation of DNMT1 leads to hypomethylation of the *CD47* promoter and increases the expression of CD47, a key inhibitor of macrophage phagocytosis. Elevated CD47 expression suppresses macrophage phagocytosis and promotes the survival of TMZ‐treated GBM cells. The small molecule WIN 51708 disrupts the p‐GSK3β‐Y216‐DNMT1 interaction, which stabilizes DNMT1, decreases CD47 expression, restores phagocytosis in vivo, and resensitizes tumors to TMZ. The GSK3β‐DNMT1‐CD47 axis was found to be conserved in a TMZ‐resistant PDX model and in samples from patients with recurrent GBM, supporting its clinical translational value. Our findings underscore the potential of the combined administration of WIN 51708 and TMZ as a strategy to resensitize GBM tumors.

## Graphical abstract

Temozolomide treatment activates GSK3β, driving DNMT1 phosphorylation, destabilization, and CD47 promoter hypomethylation in glioblastoma. This epigenetic shift upregulates CD47, enabling TMZ‐treated GBM cells to evade macrophage phagocytosis, survive chemotherapy, and acquire resistance. Brain‐penetrant WIN 51708 disrupts the GSK3β‐DNMT1 axis, restores macrophage‐mediated phagocytosis, and re‐sensitizes tumors to temozolomide.

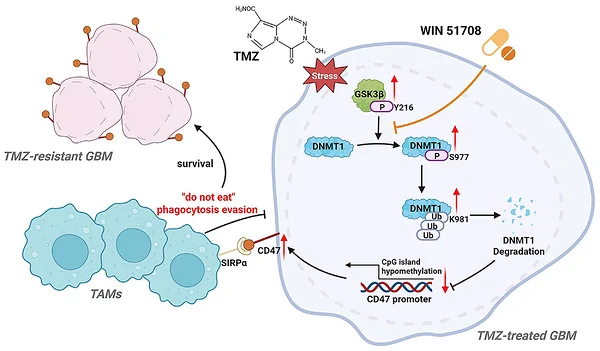

## 1 Introduction

Glioma tumors are the most common primary malignant tumors of the central nervous system and are associated with poor outcomes [1]. Glioblastoma (GBM), the most aggressive subtype of glioma, accounts for approximately half of all glioma cases [2]. The current standard of care for GBM patients involves radiotherapy in combination with temozolomide (TMZ), which increases median survival from 12.1 to 14.6 months [3, 4]. However, nearly 90% of GBM patients experience recurrence in situ after initial treatment, and many recurrent tumors exhibit acquired resistance to TMZ [5, 6].

GBM cells readily acquire resistance through multiple mechanisms, including increased O6‐methylguanine‐DNA methyltransferase (MGMT) activity [7], increased plasma membrane localization of ABCB1 [8], and maintenance of reactive oxygen species (ROS) homeostasis [9], among other factors [10, 11]. Moreover, TMZ treatment reshapes the tumor microenvironment (TME), and the cross talk between GBM cells and other cell populations contributes to GBM recurrence and TMZ resistance [12, 13]. Among these cell populations, tumor‐associated macrophages (TAMs), the predominant immune cells in the GBM TME, have been implicated in TMZ resistance [14, 15], largely because of their M2‐like phenotype [16]. However, the underlying mechanism is not fully understood. Specifically, whether TAM phagocytosis plays a role in the development of TMZ resistance is unclear.

Macrophage‐mediated phagocytosis is a crucial innate immune mechanism for the elimination of tumor cells [17]. This process is regulated by a balance of prophagocytic (“eat‐me”) and antiphagocytic (“don't‐eat‐me”) signals present on the surface of tumor cells [18]. Notably, the CD47‐signal regulatory protein α (SIRPα) axis functions as a primary inhibitory checkpoint, allowing tumor cells to evade macrophage‐mediated phagocytosis [19, 20]. Recent studies have linked CD47 to chemoresistance in most tumors [21, 22, 23, 24]. However, whether CD47 contributes to TMZ resistance in the context of GBM has not yet been reported. Despite encouraging preclinical and early clinical data, systemic CD47‐targeting strategies face clinical limitations because of the required balance between efficacy and toxicity [25]. Therefore, clarifying the molecular mechanisms that cause the upregulation of CD47 expression, especially those involved in drug treatment, could lead to more selective and context‐dependent treatment strategies. CD47 expression is known to be controlled by protein polyubiquitination and degradation [26, 27], transcription factors [23, 28], and noncoding RNAs [29], among other factors. However, whether CD47 promotes TMZ resistance itself, whether CD47 participates in the development of resistance by inhibiting the phagocytic function of TAMs and the mechanism by which TMZ treatment increases CD47 expression remain unclear.

In this study, we demonstrate that prolonged TMZ exposure leads to the upregulation of CD47 expression in GBM cells, which suppresses TAM‐mediated phagocytosis and thereby promotes tumor cell survival and acquired resistance. Prolonged exposure to TMZ activates GSK3β, which binds to DNA methyltransferase 1 (DNMT1), leading to the phosphorylation of DNMT1 at serine 977 (S977), its ubiquitination at lysine 981 (K981), and ultimately, its destabilization. This process results in hypomethylation of the *CD47* promoter and an increase in CD47 levels. The small molecule WIN 51708 disrupts the p‐GSK3β‐Y216‐DNMT1 interaction, thereby restoring DNMT1 expression, reducing CD47 expression, restoring phagocytosis, and resensitizing tumors in vivo. The GSK3β‐DNMT1‐CD47 axis is preserved in resistant patient‐derived xenograft (PDX) and samples from patients with recurrent GBM, suggesting that targeting upstream of CD47 regulation may serve as an effective therapeutic strategy for GBM patients experiencing recurrence and treatment resistance.

## 2 Results

### 2.1 TMZ‐Induced Macrophage Infiltration in the TME Promotes GBM Chemoresistance

**FIGURE 1:**
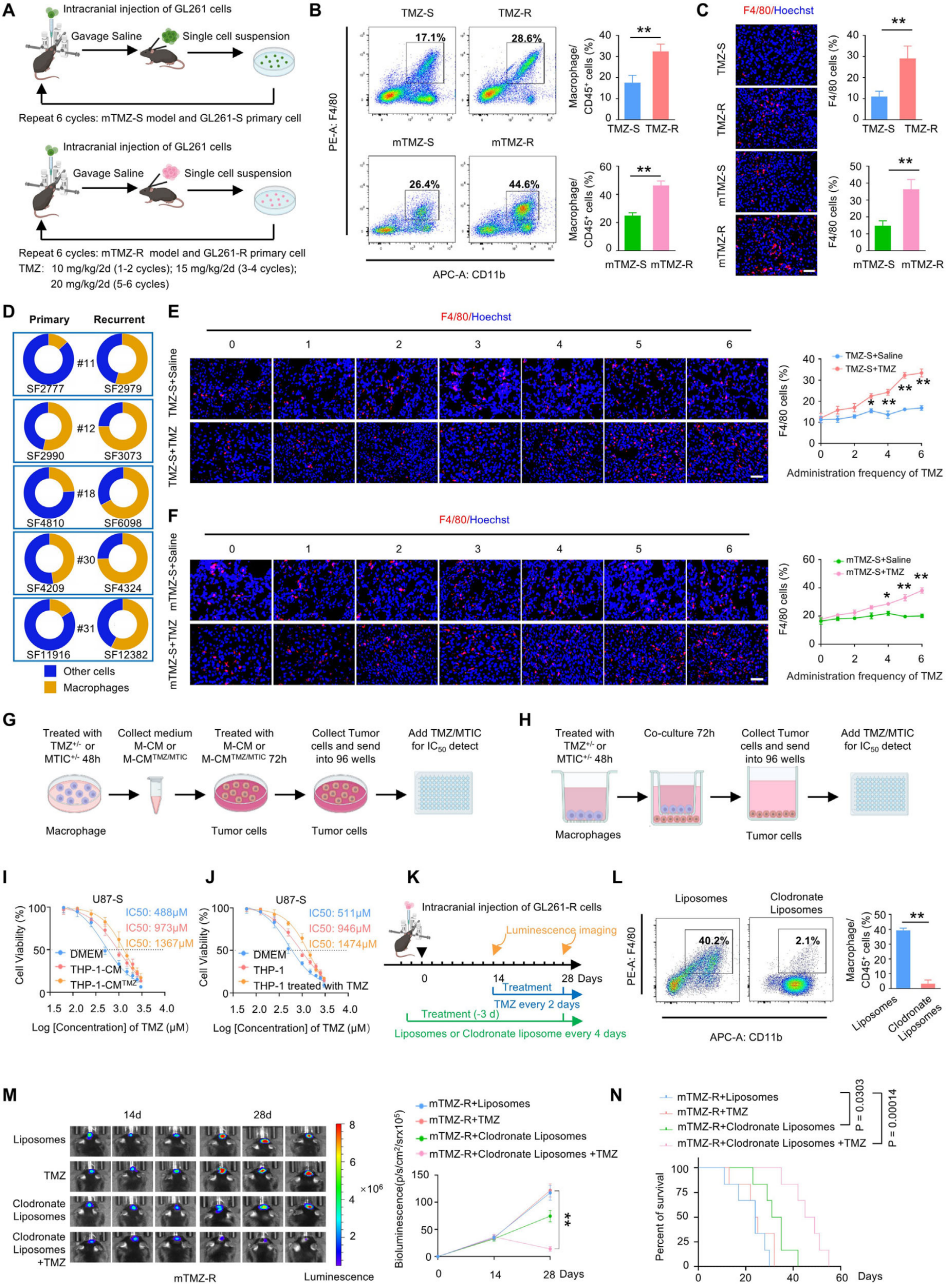
FIGURE 1 TAM accumulation promotes acquired TMZ resistance in GBM. (A) Schematic illustration of the establishment of TMZ‐sensitive (mTMZ‐S) and TMZ‐resistant (mTMZ‐R) intracranial GBM models in GL261 backgrounds. This panel was generated with BioRender.com. (B) Flow cytometry analysis (left) and quantification (right) of TAMs in TMZ‐S/R and mTMZ‐S/R models. (*n* = 3, **p < 0.01, unpaired *t‐*test). (C) Immunofluorescence (IF) staining of TAMs in TMZ‐S/R and mTMZ‐S/R models. (*n* = 3, **p < 0.01, unpaired *t*‐test; Scale bars, 200 µm.). (D) Representative pie plots showing the TAMs proportion in all cells grouped by paired primary and recurrent GBM from the GSE174554 dataset. (E‐F) IF staining of TAMs in TMZ treatment TMZ‐S (E) and mTMZ‐S models (F), with quantification on the right. (*n* = 3, **p < 0.01, one‐way ANOVA; Scale bars, 200 µm). (G) Schematic illustration of the contribution of conditioned medium derived from macrophages to GBM chemotherapy resistance. (H) Schematic diagram of the Transwell co‐culture system for assessing the contribution of macrophages to GBM chemotherapy resistance through indirect macrophage‐tumor cell interaction. (I) IC_50_ curves of TMZ response in U87‐S cells in indicated conditional medium. (J) IC_50_ curves of TMZ response in U87‐S in indicated co‐culture system. (K) Schematic of the clodronate liposome mediated macrophage depletion model. Mice were intracranially injected with GL261‐R cells and treated with clodronate liposomes or liposomes (green arrow), followed by seven doses of TMZ (20 mg·kg^−^^1^) via oral gavage starting on day 14 post‐implantation (blue arrow). These panels were generated with BioRender.com. (L) Flow cytometry analysis (left) and quantification (right) of TAMs in mouse models (panel K). (n = 3, **p < 0.01, unpaired *t*‐test). (M) In vivo bioluminescent imaging (left) and quantification (right) show the effect of clodronate liposome mediated macrophage depletion on the response of resistant tumors to TMZ. (*n* = 6, **p < 0.01, one‐way ANOVA). (N) Kaplan‐Meier survival curves of mice bearing intracranial mTMZ‐R GBM treated with TMZ in combination with control liposomes or clodronate liposomes. (*n* = 6, Log‐rank test).

To assess whether chemotherapy induced alterations in TAMs contribute to GBM resistance to TMZ, two in vivo intracranial models of TMZ resistance were established: a human‐derived model (designated TMZ‐S/R) and a mouse‐derived model (designated mTMZ‐S/R). The primary tumor cells isolated from these models were designated U87‐S/R and GL261‐S/R, respectively (Figure 1A and Figure S1A). Compared with their sensitive counterparts, resistant tumors exhibited increased intracranial growth and a greater tumor burden (Figure S1B,C). The resistant cells also presented elevated IC_50_ values for TMZ and its active metabolite 5‐(3‐methyltriazen‐1‐yl)‐imidazole‐4‐carboxamide (MTIC) (Figure S1D–G), confirming the successful establishment of GBM models with acquired resistance to TMZ. Flow cytometry and immunofluorescence (IF) staining demonstrated a significant accumulation of TAMs in TMZ‐R and mTMZ‐R tumors (Figure 1B,C). In alignment with these observations, single‐nucleus RNA sequencing revealed that recurrent GBM specimens exhibited a greater macrophage fraction than matched primary tumors did, providing orthogonal human validation of the role of TAM accumulation in TMZ resistance (Figure 1D and Figure S1H). Additionally, in the model with continuous TMZ administration, TAM infiltration increased progressively in a TMZ‐dependent manner (Figure 1E,F and Figure S1I). Coculture systems were established to assess the functional contribution of TAMs to chemoresistance (Figure 1G,H). Macrophage‐derived conditioned medium increased the subsequent resistance of GBM cells to TMZ or MTIC treatment. This effect was enhanced with conditioned medium collected from macrophages pre‐exposed to TMZ or MTIC (Figure 1I and Figure S1J–L). Consistent with these findings, macrophages promoted GBM cell resistance to TMZ or MTIC in the macrophage‐tumor coculture system, and this effect was more pronounced when macrophages were pre‐treated with TMZ or MTIC (Figure 1J and Figure S1M–O). In the mTMZ‐R mouse model, compared with TMZ plus control liposomes treatment, clodronate liposome‐mediated macrophage depletion increased the therapeutic efficacy of TMZ, as evidenced by a significantly reduced bioluminescent tumor burden and prolonged mouse survival (Figure 1K–N). These findings provide direct evidence that TAMs serve as crucial mediators of acquired resistance. Our results highlight the chemotherapy‐induced infiltration of TAMs as a causal driver of TMZ resistance in GBM tumors.

### 2.2 CD47 Upregulation is Associated with Impaired Macrophage‐Mediated Phagocytosis in TMZ‐Resistant GBM Tumors

**FIGURE 2:**
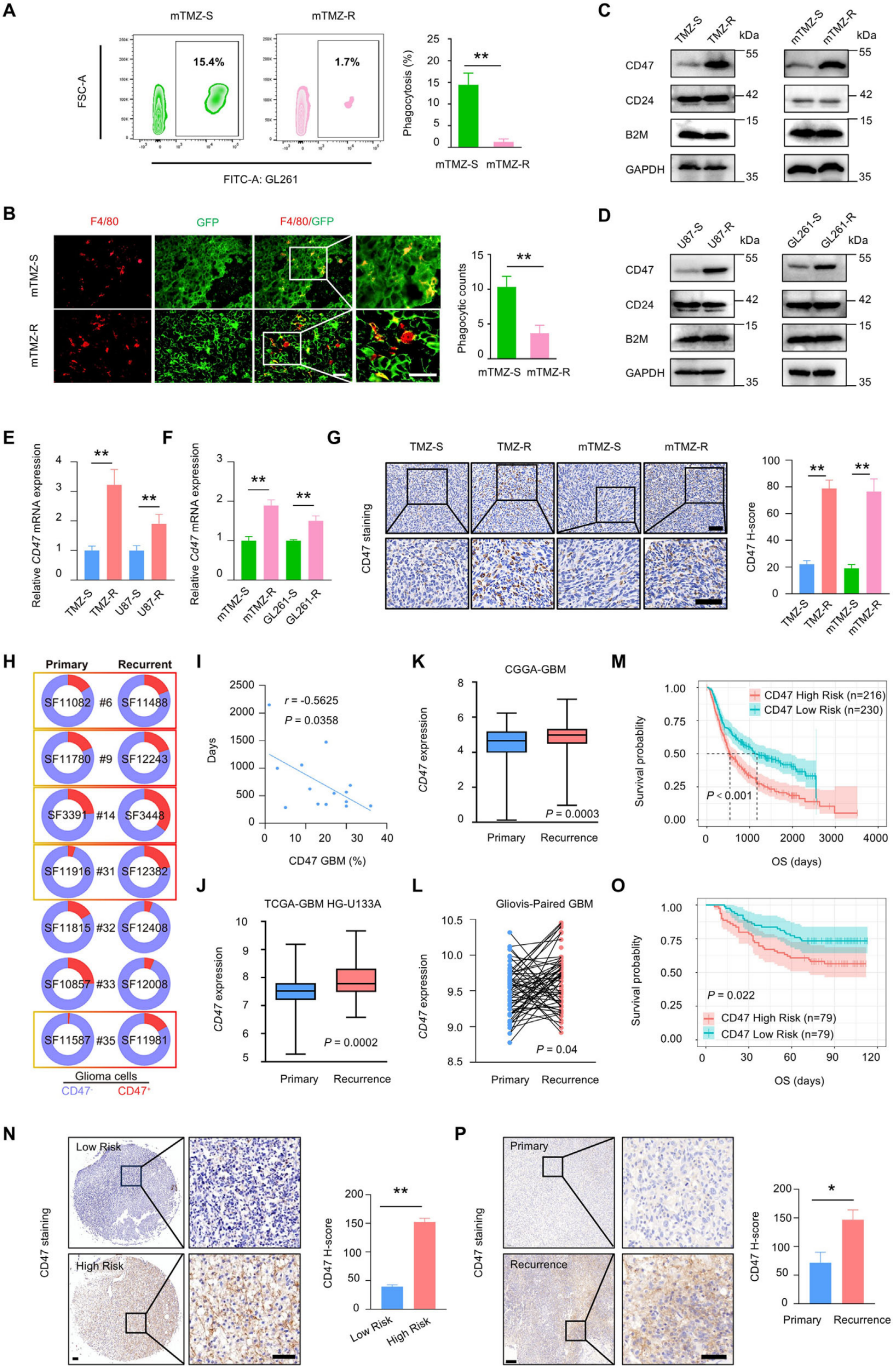
CD47 upregulation associates with impaired TAM phagocytosis in TMZ‐resistant GBM. (A) Flow cytometry‐based phagocytosis in mTMZ‐S versus mTMZ‐R tumors. Representative gating plots (left) and quantification (right). Gating strategy in Figure S2A. (n=3, **p < 0.01, unpaired *t‐*test). (B) Double IF staining (left) and quantification (right) of F4/80 and GFP in mTMZ‐S and mTMZ‐R tumors. (*n* = 3, **p < 0.01, unpaired *t‐*test, Scale bars, 200 µm). (C‐D) Representative image of CD47, CD24, and B2M protein in tumor tissues (C) and primary cells (D) isolated from TMZ‐S/TMZ‐R and mTMZ‐S/mTMZ‐R models. (E, F) *CD47* mRNA levels in tumor tissues (E) or primary cells (F) isolated from TMZ‐S/TMZ‐R and mTMZ‐S/mTMZ‐R models. (*n* = 3‐5, **p < 0.01, unpaired *t‐*test). (G) Immunohistochemistry (IHC) of CD47 in indicated tissues with representative images (left) and H‐score quantification (right). (*n* = 3, **p < 0.01, unpaired *t*‐test, Scale bars, 200 µm.). (H) Representative pie plots showing the proportion of *CD47* ^+^ cell population in glioma cells grouped by paired primary and recurrent GBM from the GSE174554 dataset. (I) The correlation analysis of *CD47* ^+^ percentage and survival days in GBM patients from GSE174554. (*p* = 0.0358, *r* = ‐0.5625, Spearman correlation). (J) *CD47* expression was compared between primary (n = 225) and recurrent (n = 133) GBM in the CGGA database. (*p* = 0.003, unpaired *t‐*test). (K) Comparison of *CD47* expression between primary (n = 497) and recurrent (n = 23) GBM in TCGA HG‐U133A database. (*p* = 0.002, unpaired *t‐*test). (L) Paired transcriptomic analysis of *CD47* expression from the GlioVis platform (n = 61). (*p* = 0.004, paired *t‐*test). (M) Kaplan‐Meier survival plot for GBM patients with high (*n* = 216) and low (n = 230) *CD47* expression from CGGA data (*p* < 0.001, Log‐rank test). (N) IHC of a commercial GBM tissue microarray (*n* = 158) showing representative CD47 staining (left) and H‐score quantification (right). (**p < 0.01, Scale bars, 100 µm). (O) Kaplan‐Meier survival plot for high risk (*n* = 79) and low risk (*n* = 79) GBM spamles. (*p* = 0.022, Log‐rank test). (P) IHC of primary (*n* = 6) and recurrent (*n* = 3) GBM tissues with representative CD47 staining (left) and H‐score quantification (right). (Scale bars, 100 µm, *p < 0.05, unpaired *t‐*test).

To explore whether the phagocytic function of TAMs is impaired in TMZ‐resistant GBM tumors, we conducted flow cytometry and IF staining using samples from the mTMZ‐S and mTMZ‐R mouse models. Our findings revealed a significant decrease in tumor cell phagocytosis by TAMs in the mTMZ‐R model compared with that in the mTMZ‐S model (Figure 2A and Figure S2A), which was confirmed by quantifying the IF staining intensity in situ (Figure 2B). We then examined the expression of the phagocytosis‐related checkpoints CD47, CD24, and B2M in our GBM models and their derivative cells. Western blotting and qPCR revealed significant upregulation of CD47 expression in TMZ‐resistant tumors and cells compared with that in their sensitive counterparts in both human‐ and mouse‐derived intracranial models, while CD24 and B2M expression did not increase consistently (Figure 2C–F and Figure S2B,C). Immunohistochemical (IHC) analysis confirmed the elevated CD47 protein expression in resistant tumors (Figure 2G). Analysis of a representative dataset of paired GBM samples with the single‐nucleus RNA sequencing data revealed an expanded *CD47* ^+^ malignant cell subset in recurrent tumors compared with that in their matched primary tumors (Figure 2H), and a greater burden of *CD47* ^+^ malignant cells was associated with shorter survival (Figure 2I). Bulk transcriptomic analysis revealed increased *CD47* expression in recurrent GBM tumors across the TCGA and CGGA cohorts (Figure 2J,K), which was validated in paired tumors from the GlioVis dataset (Figure 2L). Prognostic analyses of the CGGA data indicated that high *CD47* expression was associated with shorter overall survival (Figure 2M). This association was corroborated by IHC analysis of a commercially available GBM tissue microarray, which revealed elevated CD47 levels in tumors from patients with unfavorable outcomes (Figure 2N,O). Furthermore, an IHC evaluation of an independent cohort from our institution revealed elevated CD47 expression in tumors from recurrent GBM patients than in primary GBM tumors (Figure 2P). Assessment of the upregulation of CD47 following prolonged exposure to TMZ was performed to investigate its induction under chemotherapeutic stress in intracranial treatment models, and both the mRNA and protein levels of CD47 increased over time (Figure S2D–F). Exposure to TMZ and MTIC also led to sustained CD47 expression in chemotherapy‐sensitive GBM cell lines (Figure S2G–L). Notably, in the context of TMZ‐resistant GBM, the expression of the phagocytosis‐related receptors SIRPα (CD172a) and MERTK on TAMs was not markedly altered (Figure S2M,N). These findings indicate that TMZ resistance in GBM tumors is associated with impaired TAM‐mediated phagocytosis and the upregulation of CD47 expression in response to TMZ exposure. CD47 was therefore identified as a potential modulator of phagocytic suppression and warrants further investigation.

### 2.3 GBM Cells with High Expression of CD47 Evade Macrophage‐Mediated Phagocytosis to Survive, Leading to TMZ Resistance

**FIGURE 3:**
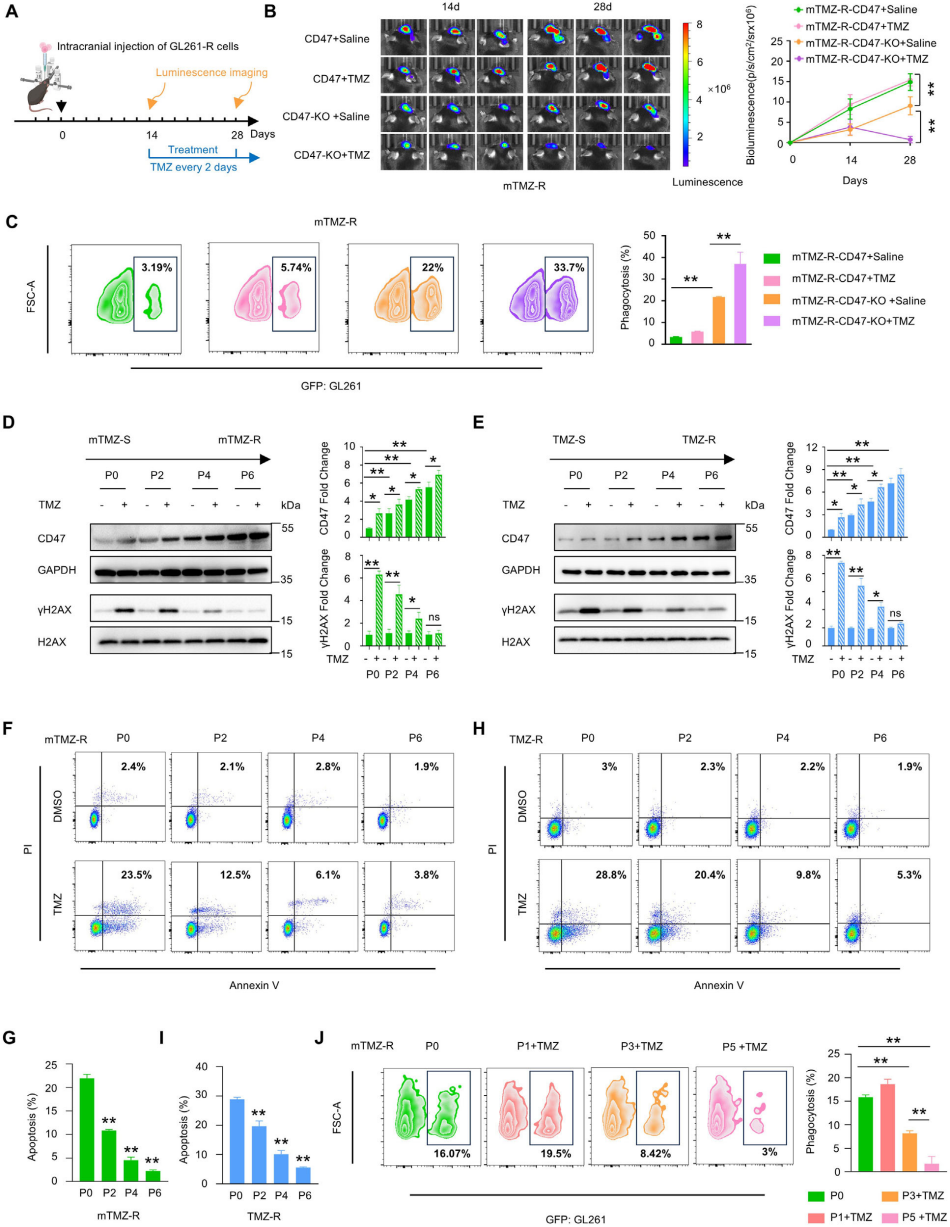
CD47 promotes macrophage evasion and persistence of TMZ‐resistant GBM under TMZ treatment. (A) Schematic illustration of the establishment of CD47 and CD47‐KO intracranial mTMZ‐R models. This panel was generated with BioRender.com. (B) Bioluminescent imaging (left) and quantification (right) of tumor growth in CD47 and CD47‐KO mTMZ‐R models treated saline or TMZ. (**p < 0.01, one‐way ANOVA). (C) Flow cytometry analysis (left) and quantification (right) of phagocytic macrophages (GFP^+^CD45^+^CD11b^+^F4/80^+^) in indicated groups. (*n* = 3, *p < 0.05, **p < 0.01, one‐way ANOVA). (D, E) Western blot analysis (left) and quantification (right) of CD47 and γH2AX expression in response to TMZ treatment tumors in the process of mTMZ‐R (D) and TMZ‐R (E) construction. P0, P2, P4, and P6 refer to primary fresh tumor cells extracted by specified algebra. (*n* = 3, *p < 0.05, **p < 0.01, two‐way ANOVA). (F) Flow cytometry analysis of apoptosis (Annexin V^+^) in response to TMZ treatment in P0, P2, P4 and P6 derived from mTMZ‐R groups. (G) The quantification of panel F. (*n* = 3, **p < 0.01, one‐way ANOVA). (H) Flow cytometry analysis of apoptosis in response to TMZ treatment in P0, P2, P4, and P6 derived from TMZ‐R groups. (I) The quantification of panel H. (n=3, **p < 0.01, one‐way ANOVA). (J) Flow cytometry analysis of macrophage‐mediated phagocytosis in indicated fresh tumors. (n=3, **p < 0.01, one‐way ANOVA). The P1+TMZ, P3+TMZ, and P5+TMZ groups refer to the orthotopic tumors of passages P1, P3, and P5 after treatment with TMZ.

Although upregulated CD47 expression is associated with phagocytosis evasion and the development of TMZ resistance, whether CD47 expression on GBM cells directly contributes to TMZ resistance remains unclear. *CD47*‐overexpressing (CD47‐OE) and *CD47*‐deficient (CD47‐KD/KO) cells were established, and their altered expression of CD47 was confirmed through Western blotting (Figure S3A,B). IC_50_ assays and chemotaxis assays revealed no significant changes in chemoresistance or macrophage migration in the context of altered CD47 expression (Figure S3C–J). These results suggest that CD47 does not directly drive TMZ resistance through a tumor cell‐intrinsic change in drug sensitivity. We therefore investigated whether CD47 contributes to TMZ resistance in vivo by protecting GBM cells from macrophage‐mediated clearance. In the mTMZ‐R CD47‐KO model, compared with control treatment, CD47 knockout markedly reduced the bioluminescent tumor burden, and TMZ further increased tumor suppression in the absence of CD47 (Figure 3A,B). Flow cytometry analyses elevated increased macrophage‐mediated phagocytosis in resistant CD47‐KO tumors, with the strongest phagocytic response observed after combined CD47 knockout and TMZ treatment (Figure 3C). These findings suggest that CD47 may promote the persistence of TMZ‐resistant tumors primarily by limiting macrophage‐mediated phagocytosis.

We then investigated whether CD47 confers selective advantages during treatment by facilitating the evasion of macrophage‐mediated clearance. Tumor cells (P2, P4, and P6) were dissociated from each generation of the intracranial TMZ‐resistant models and subjected to TMZ and MTIC treatment in vitro. Western blot analysis revealed a gradual increase in CD47 expression from P0 to P6, which was further increased by TMZ and MTIC treatment. Conversely, chemotherapy‐induced *γ*H2AX accumulation progressively decreased with increasing passage number (Figure 3D,E and Figure S4A,B). Flow cytometry analysis also revealed reduced apoptosis following TMZ and MTIC treatment, which is consistent with the acquisition of TMZ‐resistance (Figure 3F–I and Figure S4C–F). Next, we used flow cytometry to determine whether the phagocytosis of TMZ‐treated GBM cells in the microenvironment gradually decreased during the development of TMZ resistance. Compared with that in the P0 and P1 + TMZ groups, the extent of phagocytosis of level of TMZ‐treated GBM cells by TAMs gradually decreased in the P3 + TMZ and P5 + TMZ groups (Figure 3J). These results collectively highlight CD47 as a functional mediator of macrophage evasion, enabling the selection and persistence of tumor populations with TMZ‐induced high expression of CD47.

### 2.4 TMZ‐Induced Suppression of DNMT1 Expression Promotes CD47 Upregulation via Hypomethylation of Its Promoter in GBM Cells

**FIGURE 4:**
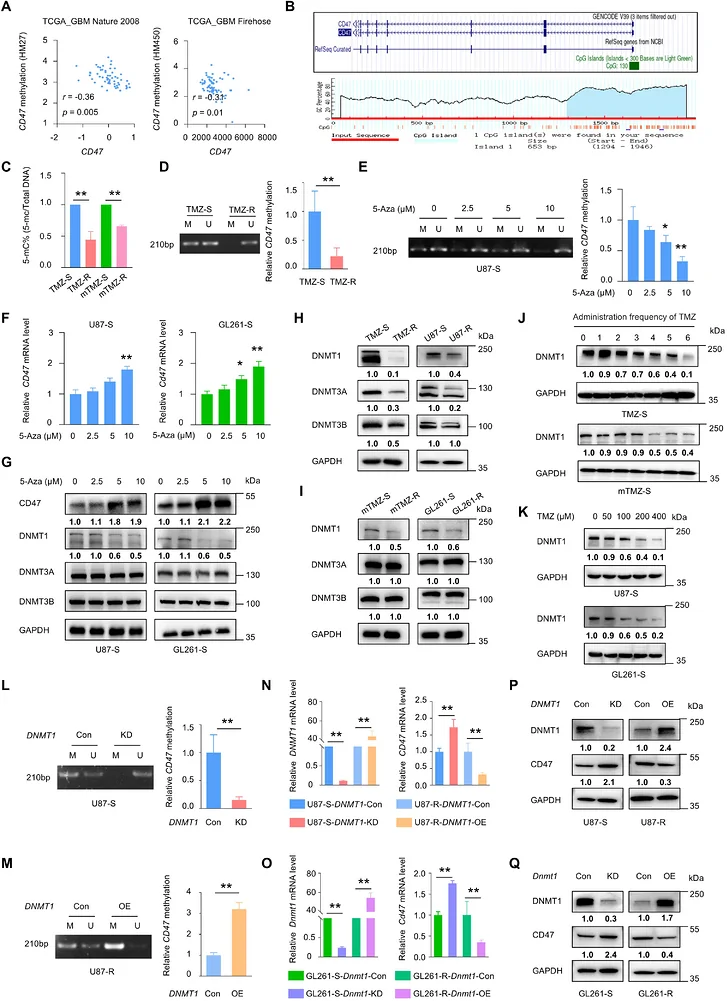
TMZ‐induced DNMT1 downregulation promotes *CD47* promoter hypomethylation and transcriptional upregulation in GBM. (A) Correlation between *CD47* mRNA level and DNA methylation M values in TCGA_GBM Nature 2008 (*n* = 58) and TCGA_GBM Firehose (*n* = 64) datasets. (B) UCSC Genome Browser view and MethPrimer prediction indicating a CpG island within the *CD47* promoter region. (C) Global DNA methylation levels measured by 5‐methylcytosine (5‐mC) ELISA in TMZ‐resistant GBM tumors. (*n* = 3, **p < 0.01, unpaired *t‐*test). (D) Methylation‐specific PCR (MSP) analysis (left) and quantification (right) of *CD47* promoter methylation in TMZ‐S and TMZ‐R tumors. (*n* = 3, **p < 0.01, unpaired *t‐*test). (E) MSP analysis (left) and quantification (right) of *CD47* promoter methylation following 5‐Aza treatment in U87‐S cells. (*n* = 3, *p < 0.05, **p < 0.01, one‐way ANOVA). (F) qRT‐PCR analysis of *CD47* expression in U87‐S (left) and GL261‐S (right) cells treated with 5‐Aza. (*n* = 3, *p < 0.05, **p < 0.01, one‐way ANOVA). (G) Western blot analysis of CD47 and DNMTs expression in U87‐S (left) and GL261‐S (right) cells treated with 5‐Aza. (H, I) Western blot analysis of DNMTs expression in indicated tissues and cells. (J) Western blot analysis of DNMT1 expression in TMZ‐treated TMZ‐S (up) and mTMZ‐S (down) tumors. (K) Western blot analysis of DNMT1 expression in TMZ‐treated U87‐S (up) and GL261‐S (down) cells. (L, M) MSP analysis (left) and quantification (right) of *CD47* promoter methylation in *DNMT1*‐knockdown (KD) U87‐S (L) cells and *DNMT1*‐overexpressing (OE) U87‐R (M) cells. (*n* = 3, **p < 0.01, unpaired *t‐*test). (N) qRT‐PCR analysis of *DNMT1* (left) and *CD47* (right) expression in *DNMT1*‐KD U87‐S and *DNMT1*‐OE U87‐R cells. (*n* = 3, **p < 0.01, unpaired *t‐*test). (O) qRT‐PCR analysis of *Dnmt1* (left) and *Cd47* (right) expression in *Dnmt1*‐KD GL261‐S and *Dnmt1*‐OE GL261‐R cells. (*n* = 3, **p < 0.01, unpaired *t‐*test). (P) Western blot analysis of DNMT1 and CD47 expression in *DNMT1*‐KD U87‐S and *DNMT1*‐OE U87‐R cells. (Q) Western blot analysis of DNMT1 and CD47 expression in *Dnmt1*‐KD GL261‐S and *Dnmt1*‐OE GL261‐R cells.

To explore the mechanism underlying TMZ‐induced CD47 upregulation and the maintenance of high CD47 expression in TMZ‐resistant GBM cells, we first analyzed the relationship between *CD47* mRNA expression and promoter DNA methylation in the TCGA GBM cohorts. This analysis revealed a negative association between *CD47* mRNA levels and DNA methylation at the CD47 promoter (Figure 4A), suggesting a possible relationship in GBM. Further analysis using the UCSC Genome Browser and MethPrimer revealed a CpG‐rich region within the *CD47* promoter (Figure 4B). Consistent with epigenetic deregulation, global 5‐methylcytosine (5‐mC) levels were reduced in TMZ‐R and mTMZ‐R tumors compared with that in sensitive control tumors (Figure 4C). Methylation‐specific PCR (MSP) revealed decreased methylation of the *CD47* promoter in TMZ‐R tumors and TMZ‐ or MTIC‐treated U87‐S cells (Figure 4D and Figure S5A,B), supporting promoter hypomethylation as a mechanism for *CD47* activation under chemotherapeutic pressure. Moreover, analysis of the TCGA dataset revealed a negative correlation between DNMT1 mRNA expression and the CD47 mRNA level and a positive correlation between DNMT1 mRNA expression and CD47 promoter methylation (Figure S5C,D). The above results suggest that the expression of *CD47* is associated with DNA promoter methylation in GBM cells. Treatment of GBM cells with 5‐azacytidine, a specific inhibitor of DNMT1, increased the mRNA and protein expression levels of CD47 by reducing *CD47* promoter methylation (Figure 4E–G). In our models, compared with TMZ‐sensitive controls, TMZ‐resistant tumors presented lower DNMT1 protein (Figure 4H,I). Consistent with these findings, exposure to TMZ led to a gradual decrease in DNMT1 levels both in vivo and in vitro (Figure 4J,K and Figure S5E). Furthermore, MSP analysis revealed that knockdown of *DNMT1* resulted in hypomethylation of the *CD47* promoter, whereas overexpression of *DNMT1* led to its hypermethylation (Figure 4L,M). Similarly, *DNMT1* knockout increased both the transcriptional and protein levels of CD47, whereas the overexpression of *DNMT1* reduced these levels (Figure 4N–Q). These findings indicate that DNMT1‐mediated hypomethylation of *CD47* in the context of TMZ resistance may underlie the upregulation of CD47 expression.

### 2.5 Proteasome‐Mediated DNMT1 Degradation Contributes to Chemotherapy‐Associated Phagocytosis Evasion in GBM

**FIGURE 5:**
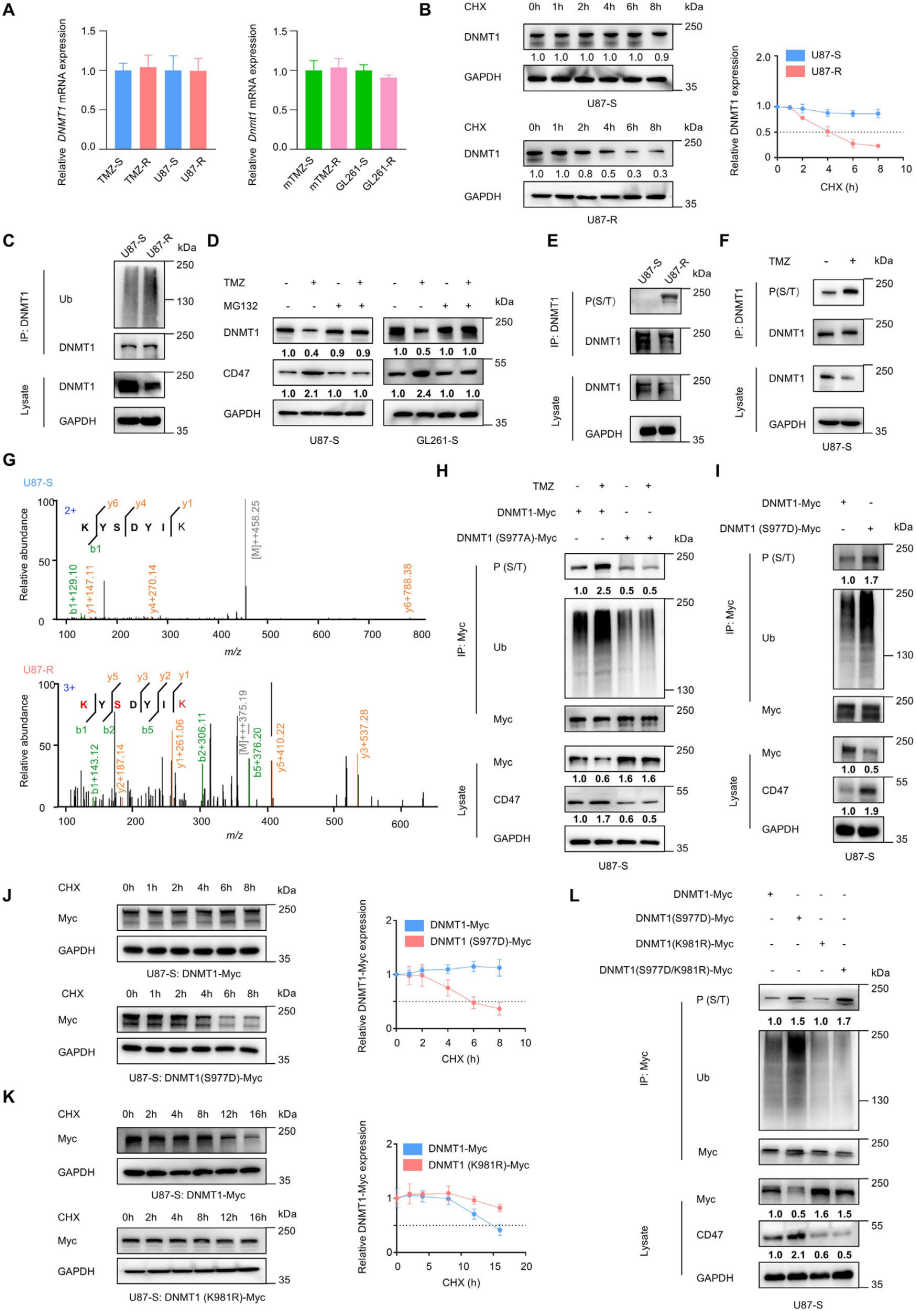
DNMT1 S977 phosphorylation promotes K981‐dependent ubiquitylation and proteasomal degradation in chemoresistant GBM. (A) qRT‐PCR analysis of *DNMT1* (left) and *Dnmt1* (right) expression in indicated tumors and cells. (*n* = 3, one‐way ANOVA). (B) Cycloheximide (CHX) assay assessing DNMT1 protein half‐life in U87‐S and U87‐R cells, with relative DNMT1 levels quantified on the right. (*n* = 3 independent experiments) (C) Immunoprecipitation (IP) analysis of DNMT1 ubiquitination in U87‐S and U87‐R cells. (D) Western blot analysis of DNMT1 and CD47 expression in TMZ and MG132 treated U87‐S (left) and GL261‐S (right) cells. (E) IP analysis of DNMT1 phosphorylation in U87‐S and U87‐R cells. (F) IP analysis of DNMT1 phosphorylation following TMZ treatment in U87‐S cells. (G) Quantitative mass spectrometry identifying serine 977 (S977) and lysine 981 (K981) as the major phosphorylation and ubiquitination sites enriched in U87‐R cells. (H) IP analysis of DNMT1 phosphorylation and ubiquitination in U87‐S cells expressing DNMT1 or DNMT1 S977A mutant, with or without TMZ treatment. (I) IP analysis of DNMT1 phosphorylation and ubiquitination in U87‐S cells expressing DNMT1 or DNMT1 S977D mutant. (J) 0‐8 h CHX chase assay comparing DNMT1 stability in U87‐S cells expressing DNMT1 or S977D mutant. Right, densitometric quantification of DNMT1 levels. (K) 0‐16 h CHX chase assay comparing DNMT1 stability in U87‐S cells expressing DNMT1 or DNMT1 K981R mutant. Right, densitometric quantification of DNMT1 levels. (L) IP analysis of DNMT1 phosphorylation and ubiquitination in U87‐S cells expressing DNMT1, DNMT1 S977D, DNMT1 K981R and DNMT1 S977D and K981R double mutants. For all CHX assays (B, J, K), cells were treated with CHX (20 µg/mL) for the indicated time before harvest.

Expanding upon the correlation between DNMT1 degradation and increased CD47 expression, we investigated the mechanism by which DNMT1 is downregulated under TMZ treatment. Despite stable *DNMT1* transcript levels, TMZ‐resistant tumors and TMZ‐exposed GBM cells showed a marked reduction in DNMT1 protein abundance (Figure 5A and Figure S6A–C). Cycloheximide (CHX) assays revealed accelerated degradation of DNMT1 in U87‐R cells (Figure 5B), and immunoprecipitation (IP) revealed increased DNMT1 polyubiquitination in these cells (Figure 5C). To determine whether DNMT1 degradation was mediated by proteasomal or lysosomal degradation, we treated U87‐S and U87‐R cells with the proteasome inhibitor MG132 or the lysosome inhibitor Lys05. Treatment with MG132, but not Lys05, restored DNMT1 levels in U87‐R cells (Figure S6D,E). Consistently, MG132 attenuated the TMZ‐ and MTIC‐induced degradation of DNMT1 and reduced CD47 upregulation in GBM cells (Figure 5D and Figure S6F), suggesting that proteasome‐mediated DNMT1 degradation contributes to the chemotherapy‐associated induction of CD47 expression and evasion of macrophage phagocytosis.

Posttranslational modifications (PTMs) influence the ubiquitination and degradation of DNMT1 [30]. IP and western blotting revealed that compared with sensitive cells, DNMT1 phosphorylation and lysine142 (K142) methylation were increased and DNMT1 acetylation was decreased in resistant cells (Figure 5E and Figure S6G,H). Following exposure to TMZ and MTIC, DNMT1 phosphorylation increased further, while acetylation and K142 methylation showed minimal changes (Figure 5F and Figure S6I–M). These findings highlight phosphorylation as the key PTM associated with DNMT1 destabilization under chemotherapeutic stress, indicating a phosphorylation‐induced, proteasome‐mediated degradation process.

### 2.6 DNMT1 S977 Phosphorylation Promotes its K981‐Dependent Ubiquitination and Proteasomal Degradation in TMZ‐Resistant GBM Cells

To identify the key phosphorylation sites responsible for the destabilization of DNMT1 in TMZ‐resistant GBM, we employed quantitative mass spectrometry. Our analysis of U87‐S and U87‐R cells revealed that S977 was one of the most highly phosphorylated sites of DNMT1 in TMZ‐resistant U87 cells (Figure 5G). We then engineered phosphodeficient (S977A) and phosphomimetic (S977D) mutants to evaluate their functions. DNMT1‐S977A reduced TMZ‐ and MTIC‐induced DNMT1 phosphorylation and polyubiquitination, which partially stabilized the protein and decreased the upregulation of CD47 (Figure 5H and Figure S6N). In contrast, DNMT1‐S977D resulted in constitutive phosphorylation and ubiquitination of this pretein, leading to DNMT1 depletion and increased CD47 expression even in the absence of treatment (Figure 5I). A 0–8 h CHX assay confirmed that compared with the wild‐type protein, DNMT1 with the S977D mutation had a shorter half‐life (Figure 5J).

Phosphorylation can activate site‐specific ubiquitination. Through quantitative mass spectrometry analysis of the DNMT1 protein, we discovered that the K981 site, which is near the S977 phosphorylation site, significantly changed in U87‐resistant cells. Consequently, we identified K981 as a potential ubiquitination site. To investigate whether K981 mediates DNMT1 ubiquitination downstream of S977, we mutated K981 to arginine (K981R). In a separate 0‐16 h CHX assay, western blot analysis revealed that K981R stabilized the DNMT1 protein following CHX treatment (Figure 5K). Importantly, co‐expression of the S977D with K981R mutations eliminated DNMT1 polyubiquitination, despite ongoing S977 phosphorylation (Figure 5L). These findings suggest that S977 phosphorylation alone is insufficient for proteasomal degradation, and K981‐dependent ubiquitination is required. Collectively, these findings support a phosphorylation‐ubiquitination cascade in which TMZ‐induced DNMT1 S977 phosphorylation enables K981‐dependent polyubiquitination. This process promotes the proteasomal degradation of DNMT1, which contributes to the increased CD47 transcription and the ability of TMZ‐resistant GBM cells to evade macrophage‐mediated phagocytosis.

### 2.7 p‐GSK3β‐Y216 Promotes DNMT1 S977 Phosphorylation and K981‐Dependent Ubiquitination in TMZ‐Resistant GBM

**FIGURE 6:**
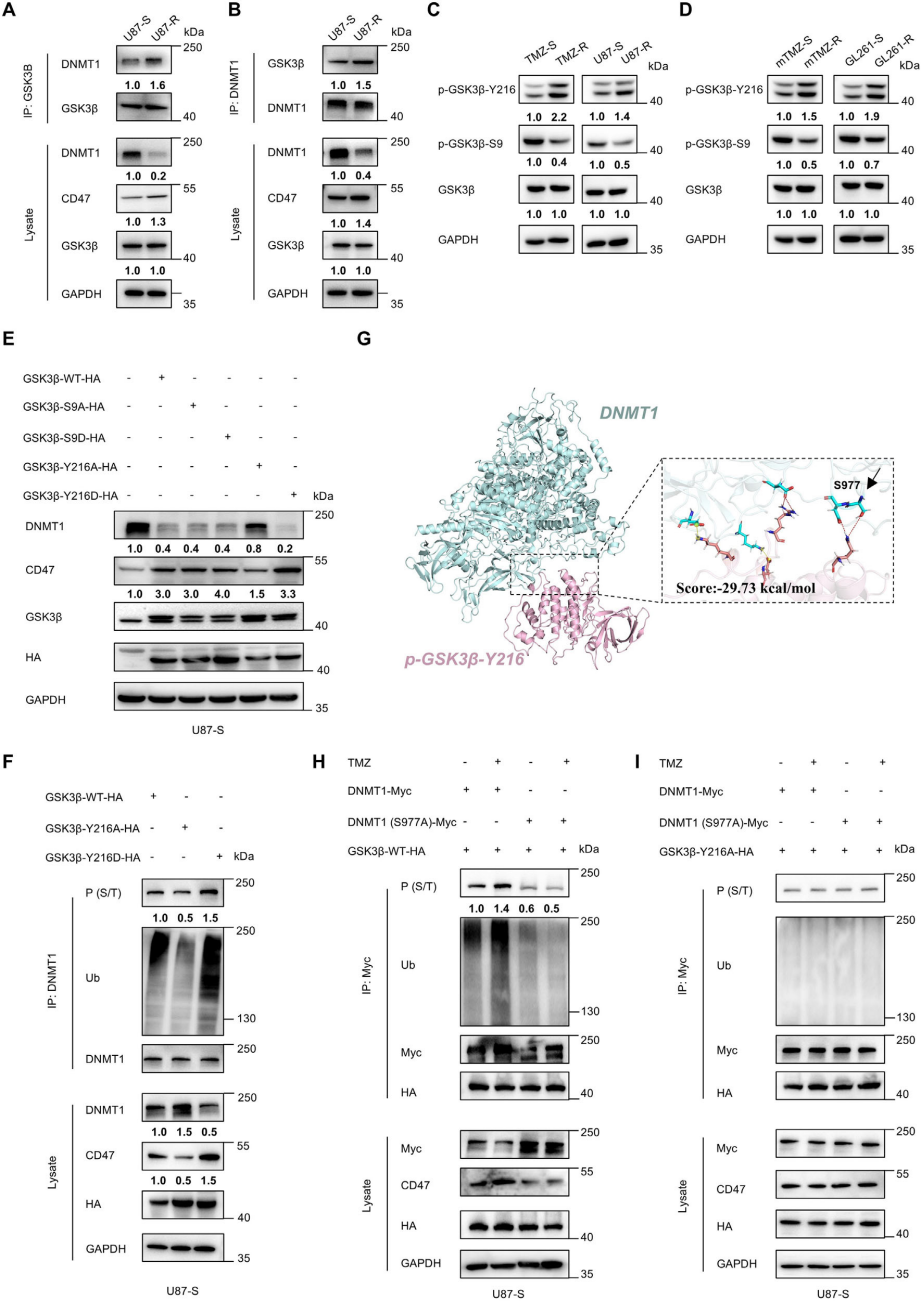
GSK3β activation promotes DNMT1 S977 phosphorylation and proteasomal degradation in chemoresistant GBM. (A‐B) IP showing interaction between GSK3β and DNMT1 in U87‐S and U87‐R cells using anti‐GSK3β (A) or anti‐DNMT1 (B) antibodies. (C) Western blot analysis of total GSK3β, its activating phosphorylation at Tyr216 (p‐GSK3β‐Y216) and inhibitory phosphorylation at Ser9 (p‐GSK3β‐S9) in TMZ‐S/R tumors (left) and U87‐S/R cells (right). (D) Western blot analysis of GSK3β and its phosphorylation states in mTMZ‐S/R tumors (left) and GL261‐S/R cells (right). (E) Western blot analysis of DNMT1 and CD47 in U87‐S cells transfected with wild‐type (WT) GSK3β or phosphorylation‐site mutants: S9A (phospho‐deficient), S9D (phospho‐mimetic), Y216A (activation‐defective) and Y216D (phospho‐mimetic) in U87‐S cells. (F) IP analysis of DNMT1 phosphorylation and ubiquitination in U87‐S cells co‐expressing DNMT1 with GSK3β‐Y216A or GSK3β‐Y216D. (G) Molecular docking model of the interaction between p‐GSK3β‐Y216 (PDB: 7Z1F) and the S977 region of DNMT1 (AlphaFold‐predicted structure). (H) IP analysis of DNMT1 phosphorylation and ubiquitination in U87‐S cells co‐expressing GSK3β‐WT with either DNMT1 or DNMT1‐S977A following TMZ treatment. (I) IP analysis of DNMT1 phosphorylation and ubiquitination in U87‐S cells co‐expressing GSK3β‐Y216A with either DNMT1 or DNMT1‐S977A, with or without TMZ treatment.

Previous work has implicated glycogen synthase kinase‐3β (GSK3β) in the regulation of DNMT1 stability via phosphorylation‐dependent mechanisms [31]. We first assessed the physical association between DNMT1 and GSK3β by performing reciprocal coimmunoprecipitation and proximity ligation assays (PLA) in U87‐S and U87‐R cells. DNMT1 and GSK3β coprecipitated under both conditions, with a stronger interaction observed in U87‐R cells (Figure 6A,B and Figure S7A), suggesting the presence of a resistance‐associated interaction. The total GSK3β abundance was comparable between sensitive and resistant cells, indicating a difference in activation state rather than protein level. Consistent with the increase in activity, p‐GSK3β‐Y216 levels increased, whereas p‐GSK3β‐S9 levels decreased in both in vitro and in vivo TMZ‐resistant models (Figure 6C,D and Figure S7B–G). Functional assays using activation‐defective (p‐GSK3β‐Y216A) and phosphomimetic (p‐GSK3β‐Y216D) variant proteins indicated that p‐GSK3β‐Y216 selectively affected DNMT1 and CD47 expression, whereas p‐GSK3β‐S9 had minimal effects (Figure 6E). Consistent with these findings, the phosphorylation and polyubiquitination of DNMT1 were increased in cells expressing p‐GSK3β‐Y216D and attenuated by the expression of p‐GSK3β‐Y216A (Figure 6F), indicating that p‐GSK3β‐Y216 is the functional form that destabilizes DNMT1 and induces CD47 expression.

In silico modeling using the crystal structure of p‐GSK3β‐Y216 (PDB: 7Z1F) and the AlphaFold‐predicted DNMT1 structure suggested a favorable interface near DNMT1 S977 (Figure 6G, binding energy −29.73 kcal mol^−^^1^), which was supported by the RMSD trajectories and MM‐GBSA scores (Figure S7H,I). Docking near K981 yielded a weaker score (−13 kcal mol^−^^1^; Figure S7J), which is consistent with the selective recognition of the S977 region. Functionally, the coexpressing GSK3β‐WT with DNMT1‐WT or DNMT1‐S977A resulted in robust TMZ‐ and MTIC‐induced of DNMT1‐WT phosphorylation and ubiquitination, but the levels of these modifications were reduced in the DNMT1‐S977A protein (Figure 6H and Figure S7K). In addition, the phosphorylation and ubiquitination of DNMT1 induced by TMZ and MTIC did not increase in U87‐S cells coexpressing p‐GSK3β‐Y216A (Figure 6I and Figure S7L). These findings support a model in which p‐GSK3β‐Y216 promotes DNMT1 S977 phosphorylation. This modification, in turn, is required for the K981‐dependent polyubiquitination and proteasomal degradation of DNMT1, thereby contributing to the induction of CD47 expression during chemotherapy.

### 2.8 WIN 51708 Disrupts the p‐GSK3β‐Y216‐DNMT1‐S977 Interaction, Stabilizes DNMT1 and Sensitizes TMZ‐Resistant GBM Cells to TMZ

**FIGURE 7:**
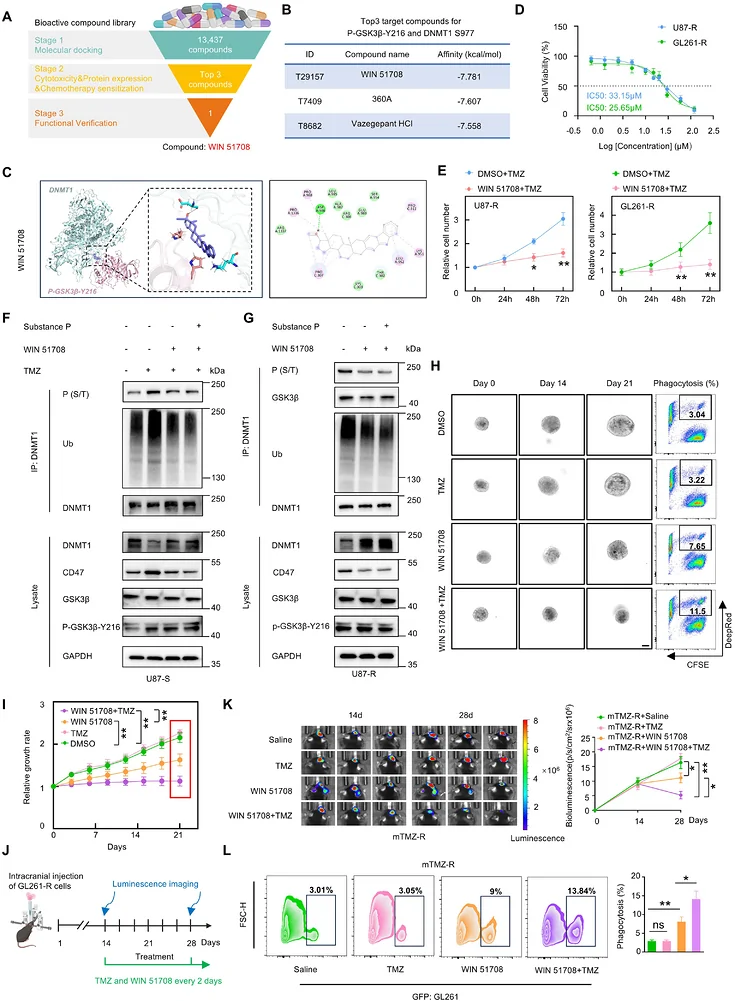
WIN 51708 disrupts the p‐GSK3β‐Y216‐DNMT1‐S977 interface to restore DNMT1 stability and overcome TMZ resistance in GBM. (A) Schematic of virtual screening steps for small molecule compounds. This panel was generated with BioRender.com. (B) The rank list of Top3 target compounds for disrupts the P‐GSK3β‐Y216 and DNMT1 S977 interaction. (C) Molecular docking model (left) and chemical structure (right) of WIN 51708 showing predicted binding at the p‐GSK3β‐Y216‐DNMT1‐S977 interface. (D) Dose‐response curves and IC_50_ determination for WIN 51708 in U87‐R and GL261‐R cells by CCK‐8 assay. (E) CCK‐8 assay measuring TMZ‐induced cytotoxicity in U87‐R (left) and GL261‐R (right) cells co‐treated with DMSO or WIN 51708 at sub‐IC_50_ concentrations. (*n* = 6, *p < 0.05, **p < 0.01, unpaired *t‐*test). (F) Western blot analysis of DNMT1 phosphorylation and ubiquitination in U87‐S cells treated with TMZ, WIN 51708 or Substance P (SP). (G) Western blot analysis of DNMT1 phosphorylation and ubiquitination in U87‐R cells treated with WIN 51708 or SP. (H) Representative image (left) and phagocytosis (right) of patient‐derived GBM organoids (PDOs) growth after combined treatment of WIN 51708 and TMZ. (Scale bars, 20 µm). (I) Growth curve of PDOs after combined treatment of WIN 51708 and TMZ. (*n* = 6, **p < 0.01, unpaired *t‐*test). (J) Schematic of combined treatment of WIN 51708 (5 mg kg^−^^1^, intraperitoneal) and TMZ (20 mg kg^−^^1^, oral gavage) in GL261‐R mouse model. (K) In vivo bioluminescent imaging (left) and quantification (right) of tumor bearing mice after combined treatment of WIN 51708 and TMZ in mTMZ‐R models. (*n* = 6, *p < 0.05, **p < 0.01, one‐way ANOVA). (L) Flow cytometry‐based in vivo phagocytosis (left) and quantification (right) in panel K. (*n* = 3, *p < 0.05, **p < 0.01, ns = not significant, one‐way ANOVA).

Building upon the role of p‐GSK3β‐Y216 in promoting DNMT1 S977 phosphorylation and K981‐dependent ubiquitination, we asked whether pharmacologic interference at this interface could restore DNMT1 stability and reduce CD47 expression. We defined a putative binding pocket at the p‐GSK3β‐Y216‐DNMT1‐S977 interface and virtually screened 13,473 drug‐like compounds from the TargetMol library. The three highest scoring candidates, WIN 51708, 360A, and vazegepant hydrochloride, exhibited favorable docking poses and interaction geometries at the S977 interface (Figure 7A–C and Figure S8A,B). All three compounds exhibited low‐micromolar activity in TMZ‐resistant GBM cells (Figure 7D and Figure S8C,D). After cotreatment with sub‐IC_50_ concentrations of these compounds and TMZ, only WIN 51708 increased TMZ‐induced cytotoxicity in U87‐R and GL261‐R cells (Figure 7E and Figure S8E,F). Mechanistically, WIN 51708 decreased DNMT1 phosphorylation and ubiquitination in TMZ‐treated U87‐S and U87‐R cells, as determined by IP and PLAs of U87‐R cells, further supporting reduced DNMT1‐GSK3β association after WIN 51708 treatment (Figure 7F,G and Figure S8G). Additionally, CHX assays revealed that WIN 51708 delayed DNMT1 degradation in resistant cells (Figure S8H), indicating that WIN 51708 stabilized DNMT1 by blocking the phosphorylation‐ubiquitination cascade.

Given that WIN 51708 is a known neurokinin‐1 receptor (NK1R) antagonist, we next investigated whether its effect on DNMT1 regulation was attributable to canonical NK1R signaling. We first examined NK1R expression in TMZ‐S and TMZ‐R GBM models. As shown in Figure S8I–L, compared with those in their sensitive counterparts, NK1R mRNA and protein levels were significantly elevated in TMZ‐resistant GBM cells and tissues. Given the increased expression of NK1R in TMZ‐R cells, we used substance P (SP), a canonical NK1R agonist, to test whether the activation of NK1R signaling could counteract the effect of WIN 51708. In TMZ‐treated U87‐S and U87‐R cells, cotreatment with WIN 51708 and SP did not reverse the WIN 51708‐induced reductions in DNMT1 phosphorylation and ubiquitination (Figure 7F,G). Consistently, in CHX chase assays, SP did not reverse the WIN 51708‐mediated stabilization of DNMT1 in resistant cells (Figure S8H). In addition, we tested aprepitant (APR), a structurally distinct NK1R antagonist, to determine whether the effect of WIN 51708 represented a general effect of NK1R antagonists. Unlike WIN 51708, APR did not produce similar inhibitory effects on DNMT1 phosphorylation or ubiquitination (Figure S8M,N). Moreover, to determine whether the TMZ‐sensitizing effect of WIN 51708 also depends on NK1R signaling, we performed cell viability assays under NK1R agonist conditions. The results showed that SP did not reverse WIN 51708‐mediated enhancement of TMZ‐induced cytotoxicity in U87‐R cells (Figure S8O). Together, these data suggest that the effect of WIN 51708 on DNMT1 phosphorylation, ubiquitination, and stability is not attributable primarily by canonical NK1R antagonism. In GBM organoids generated from patients with recurrent tumors, TMZ monotherapy was ineffective, whereas WIN 51708 alone suppressed tumor growth and, in combination with TMZ, had the strongest inhibitory effect (Figure 7H). Moreover, WIN 51708 increased the macrophage phagocytic index, with the combination of WIN 51708 and TMZ achieving the greatest increase in phagocytosis (Figure 7I).

Next, we tested the in vivo relevance of WIN 51708 with TMZ in a GL261‐R model. Notably, WIN 51708 therapy resulted in discernible antitumor activity in vivo, albeit to a lesser extent than the combination treatment did. Compared with the monotherapies, the combination treatment significantly reduced the tumor burden (Figure 7J,K). Moreover, compared with saline and TMZ alone, WIN 51708 increased the TAM phagocytic index in GL261‐R tumors, with the WIN 51708 + TMZ combination yielding the greatest increase (Figure 7L).

Collectively, these data identify WIN 51708 as a small molecule that disrupts the p‐GSK3β‐Y216‐DNMT1 interaction, resulting in DNMT1 stabilization, decreased CD47 expression, the recovery of TAM phagocytic activity, and the sensitization of TMZ‐resistant GBM cells to TMZ.

### 2.9 The GSK3β‐DNMT1‐CD47 Axis is Activated in TMZ‐Resistant PDX Models and Samples From Patients With Recurrent GBM

**FIGURE 8:**
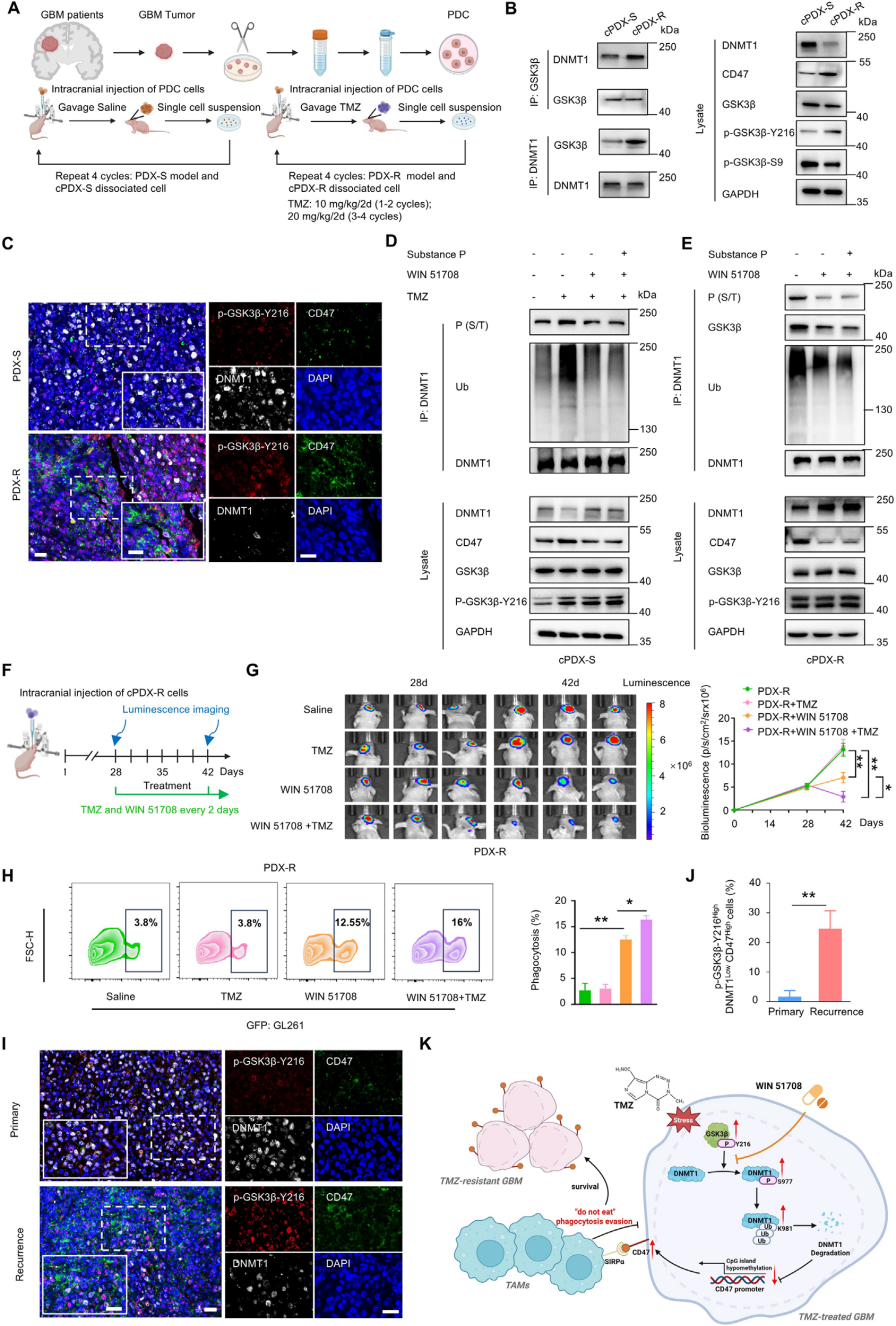
The p‐GSK3β‐Y216‐DNMT1‐CD47 axis is conserved in TMZ‐resistant PDX models and recurrent GBM tumors. (A) Schematic illustration of the establishment of PDX‐S and PDX‐R models. This panel was generated with BioRender.com. (B) Reciprocal IP showing interaction between GSK3β and DNMT1 in cPDX‐S and cPDX‐R cells using anti‐GSK3β or anti‐DNMT1 antibodies. (C) Representative images of multiplex immunofluorescence in PDX‐S and PDX‐R models. (Scale bars, 20 µm). (D) Western blot analysis of DNMT1 phosphorylation and ubiquitination in cPDX‐S cells treated with TMZ, WIN 51708 or SP. (E) Western blot analysis of DNMT1 phosphorylation and ubiquitination in cPDX‐R cells treated with WIN 51708 or SP. (F) Schematic of combined treatment of WIN 51708 (5 mg kg^−^^1^, intraperitoneal) and TMZ (20 mg kg^−^^1^, oral gavage) in PDX‐R mouse model. (G) In vivo bioluminescent imaging (left) and quantification (right) of tumor bearing mice after combined treatment of WIN 51708 and TMZ in PDX‐R models. (*n* = 6, **p < 0.01, one‐way ANOVA). (H) Flow cytometry‐based in vivo phagocytosis (left) and quantification (right) in panel G. (*n* = 6, *p < 0.05, **p < 0.01, one‐way ANOVA). I) Representative images of multiplex immunofluorescence in Primary and Recurrence GBM tumors. (Scale bars, 20 µm). J) The quantification of panel I. (*n* = 3, **p < 0.01, unpaired *t‐*test). K) The Schematic diagram of this study. This panel was generated with BioRender.com.

Next, we established a TMZ‐resistant PDX model to assess the clinical relevance of the above findings (Figure 8A). In vivo bioluminescence imaging and IC_50_ assays confirmed the successful establishment of PDX models with acquired resistance to TMZ (Figure S9A,B). MSP revealed that CD47 methylation decreased in PDX‐R tumors (Figure S9C). Furthermore, the IP and PLA results revealed a strong interaction between DNMT1 and GSK3β in both types of cells, particularly in U87‐R cells. The increased activity of GSK3β, decreased expression of DNMT1, and increased expression of CD47 were consistent with the aforementioned model (Figure 8B and Figure S9D). Multiplex immunofluorescence staining revealed that the number of p‐GSK3β‐Y216^High^ DNMT1^Low^ CD47^High^ cells increased in PDX‐R tumors (Figure 8C). These results indicate that the GSK3β‐DNMT1‐CD47 axis discovered above is conserved and reproducible in clinical samples. Moreover, WIN 51708 decreased DNMT1 phosphorylation and ubiquitination in TMZ‐treated cPDX‐S cells and cPDX‐R cells. Consistent with the results obtained in the U87 cell models, cotreatment with SP did not reverse the WIN 51708‐induced reduction in DNMT1 phosphorylation or ubiquitination in TMZ‐treated cPDX‐S and cPDX‐R cells (Figure 8D,E and Figure S9E–G). In addition, APR failed to replicate the effects of WIN 51708 on DNMT1 phosphorylation and ubiquitination (Figure S9H,I). These results further support that WIN 51708 regulates DNMT1 stability in patient‐derived GBM models through a mechanism that is not primarily explained by canonical NK1R antagonism. Like in the GL261‐R mouse model, WIN 51708 exhibited antitumor activity in the PDX‐R model. The combination of TMZ and WIN 51708 significantly reduced the tumor burden (Figure 8F,G). Phagocytosis by TAMs also increased after WIN 51708 or combination treatment (Figure 8H). Moreover, we found that the number of p‐GSK3β‐Y216^High^ DNMT1^Low^ CD47^High^ cells in the recurrent GBM samples was significantly greater than that in primary GBM samples (Figure 8I,J). Together, these data demonstrate the clinical relevance of our model to clinically relevant settings, indicating that the P‐GSK3β‐Y216‐DNMT1‐CD47 axis is activated in TMZ‐resistant PDX and recurrent GBM samples.

## 3 Discussion

TMZ treatment has been the standard care for GBM patients for more than three decades but only very moderately extends patients survival time [32]. Understanding the mechanism underlying acquired TMZ chemoresistance is therefore critical for improving treatment outcomes. In this study, we revealed that tumor cells with high CD47 expression after TMZ treatment induced GBM recurrence and resistance to TMZ by suppressing the phagocytic function of macrophages. Mechanistically, TMZ increases GSK3β activation at Y216 and promotes DNMT1 destabilization via S977 phosphorylation and K981 ubiquitination, which are associated with *CD47* promoter hypomethylation and transcriptional upregulation, thereby reducing macrophage‐mediated clearance. Pharmacologic disruption of the p‐GSK3β‐Y216‐DNMT1 interaction with WIN 51708 stabilizes DNMT1, decreases CD47 expression, restores phagocytosis in vivo, and sensitizes resistant tumors to TMZ. The activation of the p‐GSK3β‐Y216‐DNMT1‐CD47 axis in TMZ‐resistant PDXs and recurrent tumors supports its clinical relevance. This axis is associated with the evasion of macrophage‐mediated phagocytosis and chemoresistance and may represent a tractable target for therapeutic intervention in patients with recurrent disease (Figure 8K).

TMZ remains the cornerstone chemotherapeutic agent for GBM patients. However, the intrinsic and acquired resistance of GBM tumors is often an important factor contributing to disease recurrence and poor prognosis [33]. Apart from the resistance mechanisms caused by the molecular heterogeneity of GBM, acquired resistance is closely related mainly to DNA repair mechanisms, cell survival strategies, and the TME [34, 35, 36, 37]. TAMs are considered crucial factors that cause resistance to chemotherapy and radiotherapy through cell‐to‐cell communication in the context of GBM [38, 39, 40, 41]. However, few studies have investigated the dynamic changes in TAM infiltration during treatment. In this report, we utilized intracranial treatment and TMZ‐resistant GBM models and reported that the infiltration of TAMs was increased in the TMZ‐resistant GBM TME and that TMZ treatment could chemotactically increase TAM infiltration. In addition, in vitro and in vivo experiments demonstrated that macrophages contributed to the resistance of GBM cells to TMZ. This finding is consistent with previously reported results. Research on the relationship between TAMs and TMZ resistance has focused mainly on the polarization and cytotoxic effects of TAMs. Studies on the involvement of macrophage phagocytosis in the development of TMZ resistance and GBM recurrence are rare. In our study, we also detected phenotypic remodeling of TAMs in the TMZ‐R microenvironment, characterized by an increased proportion of CD206^+^ M2‐like TAMs and a reduced proportion of iNOS^+^ M1‐like TAMs (Figure S10A,B). Importantly, despite this change in TAM polarization, we found that their phagocytic function gradually decreased during the development of drug resistance. This led us to hypothesize that, during chemotherapy, increased TAM infiltration may coexist with reduced macrophage‐mediated clearance, allowing TMZ‐treated GBM cells to survive and eventually contribute to recurrence and acquired TMZ resistance. Moreover, our analysis of SIRPα and MerTK expression did not reveal marked differences between TAMs from mTMZ‐S and mTMZ‐R tumors, suggesting that the reduced phagocytic activity was not closely associated with the altered expression of these receptors alone. Interestingly, two other recent studies have shown that the decline in the phagocytic function of TAMs after chemotherapy and radiotherapy is related to myocardial injury and nasopharyngeal carcinoma recurrence, although the cells with high CD47 expression detected in these studies were tumor‐associated fibroblasts rather than tumor cells [22, 42].

Although the present study focused on TAM‐mediated phagocytic evasion, GBM progression and acquired resistance to TMZ occur within a complex stromal ecosystem in which GBM cells interact dynamically with stromal components, astrocytes, and other immune cell subpopulations [43, 44], in addition to TAMs. Astrocytes are particularly relevant in the context of GBM because they are abundant in the brain parenchyma and can be converted into a tumor‐associated astrocyte‐like states by glioma cells [45, 46]. Previous studies have shown that astrocytes can support GBM invasion, survival and therapeutic resistance through direct cell‐cell contact, soluble factors and extracellular vesicle‐mediated communication. For example, reactive astrocyte‐derived exosomes can deliver MGMT mRNA to glioma cells and confer a TMZ‐resistant phenotype, and transformed astrocytes can promote TMZ resistance by delivering ALKBH7 and increasing APNG expression [47, 48]. The extracellular matrix (ECM) is another important noncellular component of the GBM microenvironment. GBM ECM is enriched in hyaluronic acid, tenascin‐C, laminin, fibronectin, and collagen‐like matrix proteins, which regulate tumor cell invasion, stemness, mechanical adaptation, and therapeutic response [49, 50, 51]. In addition, other immune cell populations may participate in GBM progression, recurrence, and treatment resistance. For example, neutrophils and neutrophil extracellular traps (NETs) have emerged as regulators of GBM progression, and recent work has further shown that neutrophils accumulate around the surgical cavity and release NETs that promote the growth and migration of postoperative residual GBM cells [52, 53]. Moreover, a recent Cancer Cell study revealed that infiltrating plasma cells can maintain GBM stem cell programs through IgG‐tumor binding, suggesting that humoral immune components may directly influence malignant cell states associated with recurrence and therapy resistance [54]. Dendritic cells (DCs), T cells, and B‐lineage cells may also influence GBM progression, recurrence, and treatment outcome, although their direct roles in TMZ resistance remain less well defined. DCs regulate antigen presentation and T‐cell priming, providing the biological basis for DC vaccine strategies in the context of GBM, including studies that combine a vaccine with standard TMZ‐based therapy [55]. T‐cell responses in GBM are frequently constrained by exhaustion and myeloid cell‐derived suppressive signals such as IL‐10 [56]. B‐Lineage cells are emerging as functional components of the GBM immune microenvironment, although their direct contribution to TMZ resistance remains insufficiently defined [57]. These studies suggest that therapeutic pressure can reshape not only tumor‐intrinsic programs, but also the surrounding immune and stromal microenvironment, supporting the view that acquired TMZ resistance is likely driven by coordinated intercellular communication.

The CD47/SIRPα signaling axis is a key factor involved in the interaction between TAMs and tumor cells that influences phagocytic activity and is closely related to tumor immune evasion and chemoresistance [21, 23]. The main goal of these studies is to reduce the expression of CD47 in drug‐resistant cells to increase the efficacy of immunotherapy, but the relationship between the expression of CD47 in tumor cells themselves and chemotherapy resistance has not been explored. In this study, we found that high expression of CD47 in GBM cells itself did not promote chemoresistance to TMZ. However, the increase in CD47 expression after combined treatment and during the development of TMZ resistance supports the possibility that the weakened phagocytosis of TMZ‐treated GBM cells by TAMs leads to the survival of tumor cells. In the TME, in addition to TAMs, DCs and NK cells can also act as scavengers to eliminate treated tumor cells [58], but data on the infiltration and changes in the functions of these cells in TMZ‐treated GBM tumors are lacking. Moreover, the expression of CD24 [59] and B2M [60], which are associated with the phagocytic function of TAMs, in GBM was not significant.

The clinical application of CD47‐targeted therapy of CD47 is severely limited because of the potential side effects [61]. Therefore, understanding the molecular mechanisms that regulate CD47 expression for targeted therapy is crucial. In this study, we revealed that DNA hypomethylation, mediated by DNMT1 under TMZ treatment, leads to high CD47 expression in GBM cells both in vivo and in vitro. These findings reveal a new strategy for targeting CD47 in patients with recurrent and chemoresistant GBM. Although DNMT1 is downregulated in chemoresistant GBM cells and can impact GBM TMZ resistance in vitro [62], the role of DNMT1 in the formation of TMZ‐resistant GBM tumors still requires further investigation.

In this study, we observed reduced DNMT1 protein expression in TMZ‐treated and TMZ‐resistant GBM models, which was mediated by its phosphorylation‐dependent ubiquitination and subsequent proteasomal degradation. Consistent with this mechanism, TMZ activates GSK3β, a kinase that phosphorylates DNMT1 to trigger its ubiquitination and degradation [31]. Notably, in GBM, we identified that S977, rather than the previously reported residues S410/S414, as the DNMT1 residue phosphorylated by GSK3β, and we found that in our models, TMZ‐induced GSK3β activation was predominantly associated with increased phosphorylation at Y216, while consistent changes in inhibitory phosphorylation at S9 were not observed. In addition to phosphorylation [63], DNMT1 acetylation [64] and K142 methylation [65] have been implicated in the regulating DNMT1 polyubiquitination. However, following TMZ treatment, we did not detect appreciable changes in DNMT1 acetylation or K142 methylation in GBM cells. These findings suggest that phosphorylation may precede and predominate in the initiation of DNMT1 ubiquitination under these conditions, although the temporal hierarchy requires further experimental validation. In addition, we identified K981 as a previously unreported ubiquitination site on DNMT1, as it emerged as the top differential residue in our mass‐spectrometry analysis and ranked first in the in silico ubiquitination site prediction. While these findings position K981 as a key ubiquitination hotspot in this context, the specific linkage type (e.g., K48‐linked) and the cognate E3 ligase(s) remain to be defined in future studies. Because DNMT1 broadly maintains DNA methylation, we also investigated whether the GSK3β‐DNMT1 axis affects the methylation of the *MGMT* promoter, a clinically established biomarker of TMZ responsiveness in GBM [66, 67]. U87 cells have been reported to be MGMT deficient and have a methylated *MGMT* promoter [68]. Consistent with these findings, *MGMT* promoter methylation remained high in both U87‐S and U87‐R cells and in the corresponding TMZ‐S and TMZ‐R tumor tissues (Figure S10C,D). In the cPDX‐derived sensitive and resistant models, *MGMT* promoter methylation also did not differ between matched sensitive and resistant samples (Figure S10E,F). These findings suggest that, in the models examined here, activation of the GSK3β‐DNMT1‐CD47 axis is associated with *CD47* promoter hypomethylation without detectable alterations in *MGMT* promoter methylation. Future studies using additional *MGMT*‐methylated and *MGMT*‐unmethylated GBM models will be needed to determine whether this axis affects *MGMT* methylation in other epigenetic contexts.

Given that p‐GSK3β‐Y216 binds DNMT1 and promotes its phosphorylation at S977 for subsequent ubiquitination, we used molecular docking to identify molecules that may disrupt the p‐GSK3β‐Y216‐DNMT1 interface, with priority given to brain‐penetrant small molecules. Among the candidates, WIN 51708, a nonpeptide NK1R antagonist [69], emerged as the top hit. WIN 51708 has been reported to exert neuromodulatory effects in preclinical models [70, 71], whereas its anticancer activity has not been well characterized. Nonpeptide NK1R antagonists, including APR, have also been reported to cross the blood‐brain barrier and to exert antitumor effects in several cancer contexts, supporting the broader therapeutic relevance of this compound class [72, 73]. In our models, WIN 51708 disrupted the interaction between p‐GSK3β‐Y216 and DNMT1, leading to decreased DNMT1 phosphorylation and ubiquitination. This disruption further stabilized DNMT1 expression, reduced CD47 expression, restored macrophage‐mediated phagocytosis, and resensitized chemoresistant tumors to TMZ. Because WIN 51708 was originally developed as an NK1R antagonist, an important consideration is whether its activity in our GBM models simply reflects canonical NK1R blockade. Our additional NK1R‐related control experiments revealed that NK1R activation did not abrogate the effect of WIN 51708 on DNMT1 regulation and that a structurally distinct NK1R antagonist failed to reproduce the observed effect. Nevertheless, the effects of WIN 51708 may not be limited to GBM cells. Other microenvironmental components, including astrocytes, ECM‐associated niches, DCs, T cells, neutrophils, and other immune cells, should be considered. Future single‐cell and spatial analyses will be needed to determine how WIN 51708 reshapes the broader GBM microenvironment.

In summary, our study was motivated by the need to explain why TMZ often fails to offer durable benefit in GBM patients and to identify actionable, tumor‐intrinsic mechanisms that couple chemotherapy exposure to immune evasion. We define a macrophage‐phagocytosis‐centered model of acquired resistance in which prolonged exposure to TMZ activates GSK3β, induces the phosphorylation of DNMT1 at S977 and promotes the ubiquitination of DNMT1 at K981. This process reduces DNMT1 expression and epigenetically maintains CD47 expression, thereby attenuating TAM‐mediated clearance. By disrupting the GSK3β‐DNMT1 interface with the brain penetranting small molecule WIN 51708, we restored DNMT1 levels, decreased CD47 expression, restored phagocytosis in vivo, and resensitized tumors to TMZ. This approach presents an upstream strategy that complements and may alleviate some limitations associated with direct CD47/SIRPα blockade.

Collectively, these findings suggest a mechanism‐guided strategy for employing the brain penetranting small molecule WIN 51708 to increase macrophage‐mediated phagocytic clearance and counteract chemotherapy‐induced resistance in GBM tumors. By delineating the GSK3β‐DNMT1‐CD47 axis and its associated biomarker signature, our work may guide the development of rational combinations treatments that include TMZ and macrophage‐engaging immunotherapies that target phagocytosis checkpoints in GBM and potentially other solid tumors.

## 4 Experimental Section

### 4.1 Ethics Statement

All clinical samples involved in this study were de‐identified, and written informed consent was obtained. All corresponding experiments were approved by the medical ethics committee of The Affiliated Wuxi People's Hospital of Nanjing Medical University (KY25160) and were performed in accordance with the Declaration of Helsinki. All animal care and handling procedures were performed in accordance with the National Institutes of Health's Guide for the Care and Use of Laboratory Animals. All procedures were approved by the Institutional Review Board of Nanjing Medical University (No. (2023)131).

### 4.2 Clinical Samples

Eight primary and five recurrent GBM paraffin‐embedded tissue sections were provided by the Department of Pathology of the Affiliated Wuxi People's Hospital of Nanjing Medical University for histopathological analyses. Fresh GBM tissues for organoid culture were also collected from the same institution. All human samples were de‐identified, and the study was approved by the medical ethics committee of the Affiliated Wuxi People's Hospital (KY25160). Clinicopathologic information for all participants was provided in Table S1.

### 4.3 Cell Culture

All cell lines used in this study were cultured in the recommended culture medium at 37°C in 5% CO2. Cell sources and culture media are listed in Table S2. All cell lines tested negative for mycoplasma contamination annually and had valid STR profiles. For mouse models and patient‐derived GBM cell culture, fresh GBM tumor and tissues derived from surgery were collected immediately after tumor resection, washed, minced, and enzymatically dissociated.

### 4.4 Macrophage‐Conditioned Medium and Macrophage‐GBM Co‐Culture Assays

THP‐1 monocytes were cultured in RPMI‐1640 medium and differentiated into macrophage‐like cells by treatment with phorbol 12‐myristate 13‐acetate (PMA; 40 nM) for 48 h prior to experiments. After differentiation, cells were washed and the medium was replaced with complete DMEM before subsequent U87‐related experiments, to unify the culture conditions. RAW264.7 and GL261 cells were maintained in complete DMEM.

For conditioned medium assays, THP‐1‐derived macrophages or RAW264.7 cells were treated with TMZ or MTIC for 48 h. Cells were then washed twice with PBS and cultured in fresh drug‐free DMEM. The conditioned medium was collected and used to treat U87‐S or GL261‐S cells for 72 h. Tumor cells were then collected, reseeded into 96‐well plates, and treated with gradient concentrations of TMZ or MTIC for IC_50_ determination (Figure 1G).

For co‐culture assays, drug‐pretreated macrophages were washed and co‐cultured with U87‐S or GL261‐S cells in drug‐free DMEM for 72 h using Transwell inserts. Tumor cells were then collected and subjected to TMZ‐ or MTIC‐based IC_50_ assays (Figure 1H).

### 4.5 Patient‐Derived Glioma Organoid Culture and Cryopreservation

We adopted the previous method for the culture and preservation of organoids [74]. Briefly, freshly resected glioma tissues were transferred to a sterile glass dish containing H+GPSA medium (Hibernate A supplemented with 1× GlutaMax, 1× penicillin‐streptomycin, and 1× amphotericin B) and dissected under a stereomicroscope. Tumor fragments with a diameter of 200–500 µm were placed in an ultra‐low attachment 6‐well plate containing glioma organoid medium. The culture plate was placed on an orbital shaker at a rotation speed of 120 rpm in a humidified incubator at 37°C, 5% CO_2_, and 20% or 5% O_2_. Approximately 75% of the medium was replaced every 48 h by tilting the culture plate at a 45° angle and aspirating the supernatant above the organoids. For cryopreservation, organoids were incubated in short‐term glioma organoid medium supplemented with the ROCK inhibitor Y‐27632 for 1 h. Then, dimethyl sulfoxide was added, and the organoids along with the medium, were transferred to cryovials. The samples were initially frozen at −80°C and then stored in the vapor phase of liquid nitrogen for long‐term preservation. All organoids were cultured for at least 4 weeks before analysis.

### 4.6 Bioinformatics Data Mining and Analysis

mRNA expression data for *CD47* were obtained from the TCGA‐GBM and CGGA datasets via cBioPortal (https://www.cbioportal.org) and the CGGA database (http://www.cgga.org.cn), respectively. Paired primary and recurrent GBM samples were analyzed for *CD47* expression using the GlioVis platform (https://gliovis.bioinfo.cnio.es/). Correlations between *CD47* promoter methylation and its expression, between *CD47* promoter methylation and DNMT family members, and between *CD47* expression and DNMT family members were assessed using the TCGA‐GBM (Nature 2008) and TCGA‐GBM Firehose datasets, both accessed through cBioPortal. Detailed dataset information was provided in Table S3. For promoter methylation analyses, DNA methylation beta values were converted to M values using the formula M = log_2_[β/(1 − β)] before correlation analysis. Gene expression values were log_2_‐transformed as log_2_(FPKM + 1). Pearson correlation analysis was then performed between methylation M values and log_2_‐transformed gene expression values. Glioma standard‐of‐care therapy single‐nucleus RNA sequencing data were obtained from GSE174554. The seurat package of R software was used to perform single‐nucleus RNA sequencing cluster and annotation [74].

### 4.7 Animal

Mice were maintained under specific pathogen‐free conditions at 25°C with 60%–70% relative humidity and a 12 h light/dark cycle. All animal procedures were approved by the institutional animal care committee of Nanjing Medical University. Tumor growth in all intracranial GBM models was monitored using the IVIS Spectrum system and quantified with Living Image software. At designated time points or experimental endpoints, mice were euthanized, and tumors were collected for histological and molecular analyses.

TMZ‐sensitive and TMZ‐resistant intracranial GBM models: To establish intracranial GBM models, 5 × 10^5^ luciferase‐expressing U87 or GL261 cells were stereotactically implanted into the brains of four‐week‐old BALB/c nude or six‐week‐old C57BL/6 mice (Shanghai Laboratory Animal Center, China), respectively. To induce chemoresistance, tumors were serially passaged in vivo across six generations (P0‐P6). Beginning on day 14 post‐implantation, mice were randomized to receive saline or TMZ every other day for a total of seven doses. TMZ was administered at 10 mg/kg in P1‐P2, 15 mg/kg in P3‐P4, and 20 mg/kg in P5‐P6. These procedures yielded paired intracranial GBM models of TMZ sensitivity and resistance in human and mouse backgrounds, hereafter referred to as TMZ‐S/R and mTMZ‐S/R, respectively. Primary tumor cells dissociated from these tumors were designated U87‐S/R and GL261‐S/R (Figure 1A and Figure S1A).

Intracranial GBM models with TMZ treatment: 5 × 10^5^ luciferase‐expressing U87‐S or GL261‐S cells were implanted into the brains of BALB/c nude or C57BL/6 mice, respectively. Starting on day 14 post‐implantation, mice received saline or TMZ (20 mg/kg) every other day for six doses (Figure S1I).

Intracranial mouse TMZ‐R (mTMZ‐R) model with Macrophage depletion. Macrophage depletion model was established using clodronate liposomes. Briefly, 5 × 10^5^ GL261‐R cells were stereotactically implanted into the brains of six‐week‐old male C57BL/6 mice. Fourteen days after implantation, mice were randomized to receive TMZ (20 mg/kg) every other day for a total of seven doses. For macrophage depletion, clodronate liposomes (Liposoma BV) or control liposomes were administered via intravenous injection every four days, starting three days before the GL261‐R implantation and continuing throughout the treatment course (Figure 1K).

CD47‐WT and CD47‐KO mouse TMZ‐R (mTMZ‐R) models: CD47 wild‐type (CD47‐WT) and CD47‐knockout (CD47‐KO) GL261‐R cells expressing luciferase were used to generate mTMZ‐S models. A total of 5 × 10^5^ cells were stereotactically implanted into the brains of six‐week‐old male C57BL/6 mice. From day 14 post‐implantation, mice were randomized to receive saline or TMZ (20 mg/kg) every other day for a total of seven doses (Figure 3A).

TMZ‐resistant GBM patient‐derived xenograft (PDX‐R) models: TMZ‐resistant PDX models were established from GBM cells freshly resected from specimens obtained from clinical GBM patients. Briefly, we extracted single‐cell suspensions of tumor tissue from GBM patients, labeled them with luciferase after growth stabilization, and subsequently injected 1 × 10^6^ cells intracranially into the brain of four‐week‐old BALB/c nude mice. To induce chemoresistance, tumors were serially passaged in vivo across four generations (P0‐P4). Beginning on day 28 post‐implantation, mice were randomized to receive saline or TMZ every other day for a total of seven doses. TMZ was administered at 10 mg/kg in P1‐P2 and 20 mg/kg in P3‐P4. Through these methods, we obtained matched PDX models representing TMZ sensitivity and resistance, now referred to as PDX‐S/R, primary tumor cells isolated from these xenografts were named cPDX‐S/R (Figure S9A).

Combined TMZ and WIN 51708 treatment in mTMZ‐R and PDX‐R models: To evaluate combination therapy in mTMZ‐R and PDX‐R GBM models, 5 × 10^5^ luciferase‐expressing GL261‐R cells or 1 × 10^6^ luciferase‐expressing cPDX‐R were stereotactically implanted into the brains of six‐week‐old male C57BL/6 and four‐week‐old BALB/c nude mice, respectively. For the mTMZ‐R and PDX‐R models, on day 14 or 28 post‐implantation respectively, mice were randomized to receive saline, TMZ (20 mg/k, oral gavage), WIN 51708 (5 mg/kg, intraperitoneal), or the combination, administered every other day for seven doses (Figures 7J and 8F).

### 4.8 Immunohistochemistry and Multiplexed Immunofluorescence Staining

For IHC analysis, paraffin‐embedded GBM tissues were obtained from three sources: clinical specimens collected from The Affiliated Wuxi People's Hospital of Nanjing Medical University, commercial GBM tissue microarrays were purchased from Shanghai Outdo Biotech Company (Cat No. HBraG159Su01), and intracranial tumor tissues derived from TMZ‐S/R and mTMZ‐S/R models. IHC staining was performed by Wuhan Servicebio Technology Co., Ltd. (Wuhan, China), and staining intensity was quantified using the Aipathwell image analysis system to evaluate expression patterns across samples.

Multiplex Immunofluorescence staining of GBM tissues were performed performed according to our previously established protocol [74]. Briefly, tissue slides were first deparaffinized and then incubated sequentially with primary antibodies followed by horseradish peroxidase‐conjugated secondary antibody incubation and tyramide signal amplification. The slides were microwave heat‐treated after each tyramide signal amplification operation. Nuclei were stained with DAPI after all the antigens above had been labeled. The immunofluorescence images were captured via microscopy (CarlZeiss LSM880), which captures the fluorescent spectra at 20 nm wavelength intervals from 420 to 720 nm with identical exposure time. Multilayer images were imported to ZEN for quantitative image analysis. The quantities of various cell populations were expressed as the number of stained cells per square millimeter and further as the percentage of positively stained cells. Detailed information on all primary and secondary antibodies used in this study is listed in Table S4.

### 4.9 Cell Growth Inhibition Assay

Cytotoxicity studies were performed in 96‐well plates, and optimal seeding densities for each cell line were determined to ensure exponential growth during the assay. Cells were cultured with medium containing a gradient concentration of drugs for 48 h. Cell viability was determined by CCK‐8. Optical density (OD) at 450 nM was measured using a microplate reader (Thermo). The percentage of cell survival at each concentration was calculated by the formula: (OD_treated_/OD_untreated_) × 100. The IC_50_ value represented the drug concentration that reduced cell growth to 50%.

### 4.10 Macrophage Functional Assays

For Macrophage infiltration analysis, TAM infiltration was assessed using both flow cytometry and immunofluorescence staining. For flow cytometry, freshly resected murine intracranial tumors were mechanically dissociated, enzymatically digested, and filtered through a 70 µm mesh to obtain single‐cell suspensions. After blocking with 2% FBS in PBS, cells were stained with APC‐Cy7‐A‐live_Dead, PerCP‐CD45, APC‐CD11b, and PE‐F4/80 antibodies for 30 min at 4°C in the dark. Labeled cells were analyzed on a FACSCelesta flow cytometer (BD Biosciences), and data were processed using FlowJo software (v10.8.0). The proportion of TAMs was quantified as CD45^+^CD11b^+^F4/80^+^ cells within the total live‐cell population. For immunofluorescence, cryosections (20 µm) of tumor‐bearing brains were blocked with 5% donkey serum and incubated overnight at 4°C with anti‐F4/80 antibody, followed by corresponding secondary antibody. Nuclei were counterstained with Hoechst (Invitrogen), and sections were mounted with Fluoromount‐G (Southern Biotech). The percentage of F4/80^+^ cells was quantified across five randomly selected microscopic fields using ImageJ software.

### 4.11 Flow Cytometric Analysis of TAM Functional Markers

To analyze phagocytosis‐related receptors and functional polarization markers on TAMs, freshly resected murine intracranial tumors were processed into single‐cell suspensions as described above. After blocking with 2% FBS in PBS, cells were stained with eBioscience Fixable Viability Dye eFluor 780, BV480 anti‐CD45, APC anti‐CD11b, RB705 anti‐F4/80, RB722 anti‐CD172a (SIRPα), FITC anti‐CD206, and BV650 anti‐MERTK antibodies for 30 min at 4°C in the dark. For intracellular iNOS staining, cells were subsequently fixed and permeabilized, followed by incubation with PE‐Cy7 anti‐iNOS antibody according to the manufacturer's protocol. Samples were acquired on a Cytek Northern Lights spectral flow cytometer, and data were analyzed using FlowJo software. TAMs were gated as live CD45^+^CD11b^+^F4/80^+^ cells, and the expression of CD172a (SIRPα), MERTK, CD206 and iNOS was quantified within the TAM population.

For in vivo phagocytosis assay: In vivo phagocytosis was evaluated using GFP‐labeled murine and PDX intracranial tumors. Tumors were dissociated as described above, and single‐cell suspensions were stained with CD45, CD11b, and F4/80 antibodies. Phagocytosis was quantified as the percentage of CD45^+^CD11b^+^F4/80^+^GFP^+^ cells within the live‐cell population. For immunofluorescence, frozen brain sections from mice bearing GFP‐labeled GL261 tumors were stained with anti‐F4/80 and anti‐GFP antibodies. Colocalization of F4/80 and GFP signals was used to indicate macrophage‐mediated phagocytosis of tumor cells in vivo.

For in vitro phagocytosis assay: In vitro phagocytosis assays were performed using human THP‐1 monocytes and patient‐derived organoid (PDO)‐derived GBM cells. THP‐1 cells were differentiated into macrophage‐like cells by treatment with PMA for 48 h prior to experiments. Differentiated THP‐1 cells were stained with CFSE (5 µM; BD Biosciences) at 37°C for 15 min and washed three times with complete medium. GBM cells (5 × 10^5^) were labeled with CellTracker Deep Red dye (5 µM; Thermo Fisher Scientific) at 37°C for 15 min and washed with PBS containing 1% FBS. CFSE‐labeled macrophages and Deep Red‐labeled tumor cells were co‐incubated in suspension in 6‐well plates at 37°C for 1 h in a total volume of 1 mL. After incubation, cells were collected directly and washed twice with cold PBS. Phagocytosis was assessed by flow cytometry based on the frequency of double‐positive cells (CFSE^+^/Deep Red^+^), which represents macrophages that had engulfed tumor cells. Data were analyzed using FlowJo software (v10.8.0).

For macrophage chemotaxis assay: Macrophage chemotaxis was evaluated using Transwell inserts (24‐well, 8 µm pore size; Corning). In summary, 1 × 10^5^ macrophages were placed in 200 µL of serum‐free medium in the upper chamber, while the lower chamber contained 600 µL of conditioned medium from the specified cells or control medium. After 12 h at 37°C with 5% CO_2_, migrated cells in the lower chamber were stained with calcein‐AM for 20 min at 37°C, shielded from light. The cells were then rinsed with PBS and imaged using an Olympus fluorescence microscope. For quantification, calcein‐positive cells were counted in 5‐10 random fields per insert or analyzed with ImageJ, and the data were averaged across at least three biological replicates.

### 4.12 Western Blot

For western blot protein detection, cells and tumor tissues were lysed in RIPA buffer (Sigma) supplemented with protease and phosphatase inhibitor cocktails (Pierce). Equal amounts of protein lysates were resolved on 12% SDS‐PAGE gels (Vazyme) and transferred onto nitrocellulose membranes using the Novex gel transfer system (Invitrogen). Membranes were blocked and incubated overnight at 4°C with primary antibodies, followed by incubation with species‐specific HRP‐conjugated secondary antibodies (Proteintech) for 1 h at room temperature. Target bands were visualized using Tanon 5200 Multi (Tanon, China). Detailed information on the primary antibodies used for Western blot analysis was provided in Table S4.

### 4.13 Quantitative Reverse Transcription PCR (qRT‐PCR)

Total RNA from tumor cells and tissues was extracted with TRIzol reagent (Thermo‐Fisher). cDNA was synthesized using the HiScript IV All‐in‐One Ultra RT SuperMix for qPCR (Vazyme, Nanjing, China). qRT‐PCR was performed with ChamQ SYBR qPCR Master Mix (Vazyme) on a Real‐Time System (Bioer, Hangzhou, China). Data were analysis by the comparative Ct (2^−ΔΔCt^) method with amplification efficiency correction.

### 4.14 Plasmids and Primers

Plasmids, including pGV141‐EV, pGV492‐*CD47*, pGV342‐*Cd47*, pGV219‐*DNMT1*, pGV112‐*shCD47*, pGV392‐*sgCd47*, pGV348‐*GSK3β‐WT*, pGV348‐GSK3β*‐S9A*, pGV348‐GSK3β*‐S9D*, pGV348‐GSK3β*‐Y216A*, and pGV348‐GSK3β*‐Y216D* were constructed and purchased from Genechem (China). pGV219‐*DNMT1‐WT*, pGV219‐*DNMT1‐S977A*, pGV219‐*DNMT1‐S977D*, pGV219‐*DNMT1‐K981R*, and pcDNA3.1‐*Dnmt1* Plasmids were constructed and purchased from MiaoLingBio (China). *siDNMT1*, si*Dnmt1* were constructed and purchased from GENERAL BIOL (China). The primers used for qPCR and PCR are listed in Table S5. The target sequences of sh*NC*, sh*CD47*, si*NC*, si*DNMT1*, si*Dnmt1*, and sg*Cd47* are also listed in Table S5.

### 4.15 Cell Apoptosis Analysis

Flow cytometry was used to detect cell apoptosis according to the Apoptosis Detection Kit instructions (KeyGenBiotech). Briefly, cells were cultured for 24 h and incubated with TMZ (400 µM, Selleck) or MTIC (50 µM, Selleck) for another 48 h before harvest. Cells incubated with Annexin V‐Alexa Fluor647/PI were immediately analyzed by FACSCelesta flow cytometer (BD Biosciences).

### 4.16 Global DNA Methylation Assay

Global DNA methylation status was quantified by previous method [75]. Levels of 5‐methylcytosine (5‐mC) were calculated according to the manufacturer's instructions. In brief, the purified and quantified genomic DNA were bound to strip‐wells treated for high DNA affinity. The methylated fraction of the DNA was detected using capture and detection antibodies. Reaction was then quantified colorimetrically by reading the absorbance in a microplate spectrophotometer. The percentage of methylated DNA was proportional to the OD intensity measured.

### 4.17 Quantitative Methylation‐Specific PCR Assay

Genomic DNA from GBM tumors or cells were isolated using Universal Genomic DNA kit (Cwbio) and quantified using NanoDrop (ThermoFisher Scientific, Leicester, UK). Bisulfite converted DNA was used to perform the qMSP by Methylamp MS‐qPCR Fast Kit (Epigentek, Brooklyn, NY) according to the manufacturer's instructions. For each reaction, 20 ng bisulfite treated DNA was used as a template. Primers for the genes of interest were designed using MethPrimer (http://www.urogene.org/methprimer). The ACTB (𝛽‐actin) gene was used as a reference gene. No‐template controls were included in each run as negative controls. An EpiTect Control DNA, a 100% methylated DNA (Qiagen, Hilden, Germany), was used as a positive control for all genes studied. The percentage of methylated reference value (PMR) was calculated by dividing the gene/ACTB ratio in a sample by the gene/ACTB ratio in the EpiTect Control DNA (Qiagen) sample and multipling by 100. Parallel PCR reactions were carried out for the genes of interest and reference. PMR values were detected using the comparative CT method. The relationship between the percentages of methylated DNA molecules and CT is described as PMR = 2^−ΔΔCT^ × 100%.

### 4.18 Immunoprecipitation and Mass Spectrometry Analysis

IP assays were performed to investigate both protein‐protein interactions and PTMs. For interaction studies, cells were transfected with the indicated plasmids and lysed using IP lysis buffer. Whole‐cell lysates were incubated with Protein A/G magnetic beads (Bimake) conjugated with the specified primary antibodies. Immunoprecipitates were subjected to Western blot analysis to assess the binding of candidate proteins. For post‐translational modification analysis, IP was performed using an anti‐DNMT1 antibody to enrich endogenous DNMT1 from GBM cells. Subsequently, Western blot was performed using phosphorylation and ubiquitination antibodies, respectively. For the detection of the methylation level at the K142 site of DNMT1, we directly used the corresponding antibody. The identification of DNMT1 phosphorylation sites was conducted using liquid chromatography‐tandem mass spectrometry (LC‐MS/MS) analysis. This analysis was performed at Shanghai Applied Protein Technology Co., Ltd., utilizing an ultra‐high‐performance liquid chromatography system (UHPLC; 129 Infinity, Agilent Technologies) in conjunction with a quadrupole time‐of‐flight mass spectrometer (AB Sciex TripleTOF 660). The identification process was completed through database searches, as previously described. A list of all antibodies used in IP and immunoblotting was provided in Table S4.

### 4.19 Proximity Ligation Assay

PLA assay was performed using the Duolink In Situ Red Starter Kit Mouse/Rabbit (DUO92101, Sigma‐Aldrich) according to the manufacturer's protocol. Briefly, cells grown on coverslips were fixed and permeabilized, followed by blocking with Duolink Blocking Solution for 60 min at 37°C. Cells were then incubated overnight at 4°C with primary antibodies against DNMT1 and GSK3β diluted in Duolink Antibody Diluent. After washing with Wash Buffer A, cells were incubated with Duolink PLA probes for 60 min at 37°C. Ligation was then performed using Duolink ligase and ligation buffer for 30 min at 37°C, followed by amplification with Duolink polymerase and amplification buffer for 100 min at 37°C. Cells were washed with Wash Buffer B and 0.01× Wash Buffer B, mounted with Duolink In Situ Mounting Medium with DAPI, and imaged using a confocal microscope. PLA puncta were used to indicate intracellular proximity between DNMT1 and GSK3β. The primary antibody information is provided in Table S4.

### 4.20 Molecular Docking

To evaluate whether p‐GSK3β‐Y216 physically interacts with the S977 and K981 region of human DNMT1, molecular docking was performed. The amino acid sequence of human DNMT1 was obtained from the UniProt database (https://www.uniprot.org/), and its full‐length three‐dimensional structure was predicted using AlphaFold3. The predicted model was subjected to structural relaxation using the Rosetta Relax protocol, and the energy‐minimized structure was used as the receptor for docking. The structure of p‐GSK3β was retrieved from the RCSB Protein Data Bank (PDB ID: 7Z1F), and structural preprocessing‐including removal of water molecules and addition of hydrogen atoms‐was performed using PyMOL. The processed structure was exported in PDB format for subsequent calculations. Specifically, rigid‐body docking between DNMT1 and p‐GSK3β was first carried out using HDOCK to identify a plausible binding orientation. Flexible docking was then performed using the RosettaDock protocol, and resulting complexes were ranked according to Rosetta's internal scoring function. The top‐ranked models were selected for further analysis. Residue‐level interaction analysis was conducted using LigPlot+, and overall binding energy was calculated using the Interaction_Energy module in FoldX. PyMOL was used for visualization of docked conformations and interaction interfaces.

### 4.21 Small Molecule Virtual Screening

To identify small‐molecule inhibitors that potentially disrupt the interaction between p‐GSK3β‐Y216 and the S977 region of DNMT1, virtual screening was performed using a bioactive compound library from the TargetMol database (https://www.targetmol.cn), comprising 17,760 known bioactive compounds. The three‐dimensional structures of compounds were optimized using RDKit in Python. Lipinski's Rule of Five (RO5) descriptors were calculated for each compound, and molecules violating two or more RO5 rules were excluded. A total of 13,473 drug‐like compounds were retained for docking analysis. Batch conversion of ligands into PDBQT format was performed using the Raccoon interface of AutoDockTools. A genetic algorithm was used for conformational sampling, with a maximum of 10,000 evaluations per run. For each compound, 20 docking poses were generated and ranked according to their predicted binding energy scores. The top‐scoring conformation was selected for downstream analysis. The top three ranked compounds were selected for in vitro validation based on docking affinity and predicted interaction residues. Virtual screening was performed by Phadcalc company (Wuhan, China).

### 4.22 Statistical Analysis

Data are presented as mean ± standard deviation. Comparisons between two groups were performed using two‐tailed Student's *t*‐tests. For comparisons among more than two groups, one‐way ANOVA followed by Tukey's multiple‐comparisons test was used. For comparisons involving two independent variables, two‐way ANOVA followed by Tukey's multiple‐comparisons test was used. Survival analyses were conducted with the Kaplan‐Meier method and log‐rank test. Associations between variables were examined using *χ^2^* tests or Spearman's rank correlation coefficients, as appropriate. Equality of variances was tested with an F‐test. A *p*‐value < 0.05 was considered statistically significant. All statistical analyses were performed using SPSS software (v16.0; IBM), and data visualization was conducted using GraphPad Prism software (v8.0; GraphPad Software).

## Author Contributions

J.L., S.Y., and Y.Z. performed cell and mouse experiment, analyzed the data, and prepared the manuscript. J.L. and D.X. constructed tumor model. J.L., S.Y., and Y.Z. performed PCR validation and western blot. J.W., L.G., and H.L. performed Western blot and polysome profiling analysis. Q.W. provided the source of GBM tissue and study supervision. J.L. prepared the figures. Z.Y. analyzed the TCGA, CGGA, Gliovis, and GEO data. Z.P., Y.Y., and X.F. supervised the project, participated in experimental design, and provided laboratory resources.

## Funding

This work was supported by National Natural Science Foundation of China (NSFC) (82302939), Wuxi Medical Innovation Team (CXTD2021006), Program of Wuxi Medical Center, Nanjing Medical University (WMCG202517, WMCJ202403, WMCC202402), Phase II Special Fund for Scientific Research on Roentgen Imaging, Jiangsu Provincial Medical Association (SYH‐3201150‐0086 [2023033]), The Scientific and Technological Tackling (Basic Research) of TaihuLight (K20241018, K20231054), Wuxi Health Commission Youth Program (Q202322, Q202217), The Talents of Double Hundred Talent Plan (BJ2023024, HB2023017), Jiangsu Association for Science and Technology support project for young scientific and technological talents (TJ‐2023‐081), The medical Expert Team Program of Wuxi Taihu Talent Plan (THRC‐TD‐YXYXK‐2021) and The “333” Engineering Project Jiangsu Province ((2024) 3‐2416).

## Ethics Statement

All clinical samples corresponding experiments were approved by the medical ethics committee of The Affiliated Wuxi People's Hospital of Nanjing Medical University (KY25160) and were performed in accordance with the Declaration of Helsinki. All animal care and handling procedures were performed in accordance with the National Institutes of Health's Guide for the Care and Use of Laboratory Animals. All procedures were approved by the Institutional Review Board of Nanjing Medical University (No. 2023)131).

## Conflicts of Interest

The authors declare no conflicts of interest.

## Supporting information




**Supporting File**: advs77471‐sup‐0001‐SuppMat.docx.

## Data Availability

The data that support the findings of this study are available from the corresponding author upon reasonable request.
