# Supplementary material for: Chemotherapy‐Activated GSK3β‐DNMT1 Signaling Upregulates CD47 to Evade Macrophage Phagocytosis and Drive Temozolomide Resistance in Glioblastoma
Source: Adv Sci (Weinh). 2026 Aug 29:e77471. Online ahead of print. doi: 10.1002/advs.77471 (PMC13525619; doi:10.1002/advs.77471)
Supplement: Supplementary file 1 [file ADVS-9999-e77471-s001.docx]

Supporting Information

Chemotherapy-Activated GSK3β-DNMT1 Signaling Upregulates CD47 to Evade Macrophage Phagocytosis and Drive Temozolomide Resistance in Glioblastoma

Jie Li, Shuai Ying, Yi Zhang, Jingjing Wang, Quan Wan, Lingli Gong, Daxing Xu, Zhenkun Yang, Hongnian Lu, Ying Yin*, Xiangming Fang*, Zhening Pu*

**Supplementary Figure 1.**

**Figure. S1. Establishment of intracranial TMZ-resistant and treatment GBM models.** (A) Schematic illustration of the establishment of TMZ-sensitive (TMZ-S) and TMZ-resistant (TMZ-R) intracranial GBM models in U87 backgrounds. This panel was generated with BioRender.com. (B, C) Bioluminescent images (left) and quantification (right) of intracranial tumor growth in TMZ-S/R (B) and mTMZ-S/R (C) mice following TMZ treatment. (n=6, **P* < 0.05, one-way ANOVA). (D, E) IC_50_ determination of TMZ in U87-S/R (D) and GL261-S/R (E) cells. (F, G) IC_50_ determination of MTIC in U87-S/R (F) and GL261-S/R (G) cells. (H) scRNA-seq UMAP of a paired primary-recurrent GBM dataset (GSE174554). (I) Schematic of the GBM treatment model with continuous TMZ administration. Mice were intracranially injected with U87-S or GL261-S cells, followed by oral gavage of six doses of TMZ (20 mg kg⁻¹) or saline, starting on day 14 post-implantation (blue arrows indicate the dosing schedule). Tumors were collected every other day after treatment initiation for further analysis (red arrows indicate the time points of sample collection). (J) IC_50_ curves of TMZ response in GL261-S cells in indicated conditional medium. (K) IC_50_ curves of MTIC response in U87-S cells in indicated conditional medium. (L) IC_50_ curves of MTIC response in GL261-S cells in indicated conditional medium. (M) IC_50_ curves of TMZ response in GL261-S in indicated co-culture system. (N) IC_50_ curves of MTIC response in U87-S in indicated co-culture system. (O) IC_50_ curves of MTIC response in GL261-S in indicated co-culture system.

**Supplementary Figure 2.**

**Figure. S2. Gating controls and chemotherapy inducibility of CD47 in GBM.** (A) Schematic diagram of the gating strategy used in flow cytometry to quantify macrophage-mediated phagocytosis of tumor cells. (B) qRT-PCR analysis of *CD24* and *Cd24* mRNA in indicated tumors and cells. (n = 3, unpaired *t-*test). (C) qRT-PCR analysis of *B2M* and *B2m* mRNA in indicated tumors and cells. (n = 3, unpaired *t-*test). (D) qRT-PCR analysis of *CD47* mRNA in TMZ treated TMZ-S (left) and mTMZ-s (right) GBM models. (n = 3, **P* < 0.05, ***P* < 0.01, one-way ANOVA). (E, F) Western blot (top) and quantification (bottom) of CD47 in TMZ treated TMZ-S (E) and mTMZ-s (F) GBM models. (n = 3, **P* < 0.05, ***P* < 0.01, one-way ANOVA). (G) qRT-PCR analysis of *CD47* mRNA in MTIC-treated U87-S (left) and GL261-S (right) cells. (n = 3, ***P* < 0.01, one-way ANOVA). (H, I) Western blot (top) and quantification (bottom) of CD47 in MTIC-treated U87-S (H) and GL261-S (I) cells. (n = 3, ***P* < 0.01, **P* < 0.05, one-way ANOVA). (J) qRT-PCR analysis of *CD47* mRNA in TMZ-treated U87-S (left) and GL261-S (right) cells. (n = 3, ***P* < 0.01, **P* < 0.05, one-way ANOVA). (K, L) Western blot (top) and quantification (bottom) of CD47 in TMZ-treated U87-S (K) and GL261-S (L) cells. (n = 3, ***P* < 0.01, **P* < 0.05, one-way ANOVA). (M) Flow cytometric analysis (left) and quantification (right) of MERTK expression of TAMs in mTMZ-S and mTMZ-R models. (n = 3, unpaired *t-*test). (N) Flow cytometric analysis (left) and quantification (right) of CD172a expression of TAMs in mTMZ-S and mTMZ-R models. (n = 3, unpaired *t-*test).

**Supplementary Figure 3.**

**Figure. S3. CD47 expression on GBM cells does not directly confer TMZ resistance.** (A, B) Western blot analysis confirming *CD47* overexpression in TMZ-sensitive U87-S and GL261-S cells (A) and CD47 knockdown or knockout in TMZ-resistant U87-R and GL261-R cells (B). (C, D) IC_50_ determination of MTIC in *CD47*-OE (C) and CD47-deficient (D) cells. (E, F) IC_50_ determination of TMZ in *CD47*-OE (E) sensitive and CD47-deficient (F) resistant GBM cells. (G-J) Calcein staining of THP-1 (G, H) and RAW264.7 (I, J) macrophages co-cultured with indicated cells (scale bars, 200 μm, n=3, unpaired *t*-test).

**Supplementary Figure 4.**

**Figure. S4. MTIC-treated GBM cells progressively acquire CD47 upregulation and reduced DNA damage response during TMZ resistance development.** (A, B) Western blot analysis (left) and quantification (right) of CD47 and γH2AX expression in response to MTIC treatment tumors in the process of mTMZ-R (A) and TMZ-R (B) construction. P0, P2, P4, and P6 refer to primary fresh tumor cells extracted by specified algebra. (n=3, **P* < 0.05, ***P* < 0.01, two-way ANOVA). (C) Flow cytometry analysis of apoptosis in response to MTIC treatment in P0, P2, P4 and P6 derived from mTMZ-R groups. (D) The quantification of panel C. (n=3, **P* < 0.05, ***P* < 0.01, one-way ANOVA). (E) Flow cytometry analysis of apoptosis in response to MTIC treatment in P0, P2, P4 and P6 derived from TMZ-R groups. (F) The quantification of panel E. (n=3, **P* < 0.05, ***P* < 0.01, one-way ANOVA).

**Supplementary Figure 5.**

**Figure. S5. DNMT1 constrains *CD47* promoter methylation and *CD47* expression in TMZ-resistant GBM.** (A) Methylation-specific PCR (MSP) analysis (left) and quantification (right) of *CD47* promoter methylation in MTIC-treated U87-S cells. (n=3, ***P* < 0.01, unpaired *t-*test). (B) MSP analysis (left) and quantification (right) of *CD47* promoter methylation in TMZ-treated U87-S cells. (n=3, ***P* < 0.01, unpaired *t-*test). (C) Correlation between *DNMTs* and *CD47* mRNA levels in the TCGA_GBM Nature 2008 and TCGA_GBM Firehose datasets. (D) Correlation between DNMT family member expression and CD47 promoter DNA methylation M values in the indicated datasets. (E) Western blot analysis of DNMT1 expression in MTIC-treated U87-S (up) and GL261-S (down) cells.

**Supplementary Figure 6.**

**Figure. S6. Proteasome-dependent DNMT1 degradation under chemotherapeutic stress.** (A) qRT-PCR analysis of *DNMT1* expression in intracranial TMZ-treated TMZ-S (left) and mTMZ-S (right) GBM models. (n = 3, unpaired *t-*test). (B) qRT-PCR analysis of *DNMT1* expression in U87-S (left) and GL261-S (right) cells treated with MTIC. (n = 3, unpaired *t-*test). (C) qRT-PCR analysis of *DNMT1* expression in U87-S (left) and GL261-S (right) cells treated with TMZ. (n = 3, unpaired *t-*test). (D, E) Western blot analysis of DNMT1 protein expression in U87-S (D) and U87-R (E) cells treated with MG132 (10 μM), or Lys05 (5 μM) for 4 h. (F) Western blot analysis of DNMT1 and CD47 expression in MTIC and MG132 treated U87-S (left) and GL261-S (right) cells. (G) IP analysis of DNMT1 acetylation in U87-S and U87-R cells. (H) Western blot analysis of DNMT1 lysine 142 (K142) methylation in U87-S and U87-R cells. (I) IP analysis of DNMT1 phosphorylation in U87-S cells treated with MTIC. (J) IP analysis of DNMT1 acetylation in U87-S cells treated with TMZ. (K) IP analysis of DNMT1 acetylation in U87-S cells treated with MTIC. (L) Western blot analysis of DNMT1 K142 methylation in U87-S cells treated with TMZ. (M) Western blot analysis of DNMT1 K142 methylation in U87-S cells treated with MTIC. (N) IP analysis of DNMT1 phosphorylation and ubiquitination in U87-S cells expressing DNMT1 or DNMT1 S977A mutant, with or without MTIC treatment.

**Supplementary Figure 7.**

**Figure. S7. Enhanced activation of GSK3β in TMZ-treated GBM.** (A) Representative PLA (left) and quantification (right) images showing GSK3β-DNMT1 proximity signals in U87-S and U87-R cells. (n=3, scale bars, 10 μm, ***P* < 0.01, unpaired *t*-test). (B, C) Western blot analysis of total GSK3β, p-GSK3β-Y216 and p-GSK3β-S9 in intracranial TMZ-S (B) and mTMZ-S (C) GBM tumors following TMZ treatment. (D) Western blot analysis of total GSK3β, p-GSK3β-Y216 and p-GSK3β-S9 in U87-S treated with TMZ. (E) Western blot analysis of total GSK3β, p-GSK3β-Y216 and p-GSK3β-S9 in U87-S treated with MTIC. (F) Western blot analysis of total GSK3β, p-GSK3β-Y216 and p-GSK3β-S9 in GL261-S treated with TMZ. (G) Western blot analysis of total GSK3β, p-GSK3β-Y216 and p-GSK3β-S9 in GL261-S treated with MTIC. (H) Root-mean-square deviation (RMSD) trajectory of the p-GSK3β-Y216-DNMT1 complex over 100 ns of molecular dynamics simulation. (I) MM-GBSA binding-free-energy analysis indicating a stable and energetically favorable interaction between p-GSK3β-Y216 and DNMT1 at the S977 interface. (J) Molecular docking model illustrating interaction between p-GSK3β-Y216 and the K981 region of DNMT1. (K) IP analysis of DNMT1 phosphorylation and ubiquitination in U87-S cells co-expressing GSK3β-WT with either DNMT1 or DNMT1-S977A following MTIC treatment. (L) IP analysis of DNMT1 phosphorylation and ubiquitination in U87-S cells co-expressing GSK3β-Y216A with either DNMT1 or DNMT1-S977A, with or without MTIC treatment.

**Supplementary Figure 8.**

**Figure. S8. Pharmacologic profiling of secondary candidates for p-GSK3β-Y216-DNMT1 disruption and ancillary validation.** (A, B) Molecular docking model (left) and chemical structure (right) of 360A (A) and vazegepant hydrochloride (B) showing predicted binding at the p-GSK3β-Y216-DNMT1-S977 interface. (C, D) Dose-response curves and IC₅₀ determination for 360A (C) and vazegepant hydrochloride (D) in U87-R and GL261-R cells by CCK-8 assay. (E) CCK-8 assay measuring TMZ-induced cytotoxicity in U87-R and GL261-R cells co-treated with DMSO or 360A at sub-IC₅₀ concentrations. (n=3, **P* < 0.05, ***P* < 0.01, unpaired *t-*test). (F) CCK-8 assay measuring TMZ-induced cytotoxicity in U87-R and GL261-R cells co-treated with DMSO or vazegepant hydrochloride at sub-IC₅₀ concentrations. (G) Representative PLA (left) and quantification (right) images showing the effect of WIN 51708 on GSK3β-DNMT1 proximity in U87-R cells. (n=3, scale bars, 10 μm, ***P* < 0.01, unpaired *t*-test). (H) CHX chase assay showing DNMT1 protein stability in U87-R cells treated with WIN 51708 in the presence or absence of SP. (I) qRT-PCR analysis of *TACR1* mRNA levels in tumor tissues or primary cells isolated from TMZ-S and TMZ-R models. (n=3, ***P* < 0.01, unpaired *t-*test). (J) qRT-PCR analysis of *Tacr1* mRNA levels in tumor tissues or primary cells isolated from mTMZ-S and mTMZ-R models. (n=3, ***P* < 0.01, unpaired *t-*test). (K) Western blot analysis of TACR1 expression in tumor tissues or primary cells isolated from TMZ-S and TMZ-R models. (L) Western blot analysis of TACR1 expression in tumor tissues or primary cells isolated from mTMZ-S and mTMZ-R models. (M) Western blot analysis of DNMT1 phosphorylation and ubiquitination in U87-S cells treated with TMZ or Aprepitant. (N) Western blot analysis of DNMT1 phosphorylation and ubiquitination in U87-R cells treated with Aprepitant. (O) CCK-8 assay measuring TMZ-induced cytotoxicity in U87-R cells treated with WIN 51708 in the presence or absence of SP. (n=3, **P* < 0.05, ***P* < 0.01, unpaired *t-*test).

**Supplementary Figure 9.**

**Figure. S9. Establishment and characterization of TMZ-resistant PDX models.** (A) Bioluminescent images (left) and quantification (right) of intracranial tumor growth in PDX-S and PDX-R following TMZ treatment. (n=6, **P* < 0.05, one-way ANOVA). (B) IC_50_ determination of TMZ and MTIC in cPDX-S and cPDX-R cells. Data presented as mean ± s.d and were analyzed by nonlinear regression. (C) MSP analysis of CD47 promoter methylation in PDX-S and PDX-R tumors. (D) Representative PLA (left) and quantification (right) images showing GSK3β-DNMT1 proximity signals in cPDX-S and cPDX-R cells. (n=3, scale bars, 10 μm, ***P* < 0.01, unpaired *t*-test). (E) Representative PLA (left) and quantification (right) images showing the effect of WIN 51708 on GSK3β-DNMT1 proximity in cPDX-R cells. (n=3, scale bars, 10 μm, ***P* < 0.01, unpaired *t*-test). (F) qRT-PCR analysis of *TACR1* mRNA levels in tumor tissues or primary cells isolated from PDX-S and PDX-R models. (n=3, ***P* < 0.01, unpaired *t-*test). (G) Western blot analysis of TACR1 expression in tumor tissues or primary cells isolated from PDX-S and PDX-R models. (H) Western blot analysis of DNMT1 phosphorylation and ubiquitination in cPDX-S cells treated with TMZ or Aprepitant. (I) Western blot analysis of DNMT1 phosphorylation and ubiquitination in cPDX-R cells treated with Aprepitant.

**Supplementary Figure 10.**

**Figure. S10. TMZ-resistant GBM models show TAM phenotypic remodeling without detectable *MGMT* promoter methylation changes.** (A) Flow cytometric analysis (left) and quantification (right) of CD206^+^ macrophage in mTMZ-S and mTMZ-R models. (n=3, ***P* < 0.01, unpaired *t-*test). (B) Flow cytometric analysis (left) and quantification (right) of iNOS^+^ macrophage in mTMZ-S and mTMZ-R models. (n=3, **P* < 0.05, unpaired *t-*test). (C) MSP analysis (left) and quantification (right) of *MGMT* promoter methylation in U87-S and U87-R cells. (n=3, unpaired *t-*test). (D) MSP analysis (left) and quantification (right) of *MGMT* promoter methylation in TMZ-S and TMZ-R GBM tissues. (n=3, unpaired *t-*test). (E) MSP analysis (left) and quantification (right) of *MGMT* promoter methylation in cPDX-S and cPDX-R cells. (n=3, unpaired *t-*test). (F) MSP analysis (left) and quantification (right) of *MGMT* promoter methylation in PDX-S and PDX-R cells. (n=3, unpaired *t-*test).
